# A screening tool for psychological difficulties in children aged 6 to 36 months: cross-cultural validation in Kenya, Cambodia and Uganda

**DOI:** 10.1186/s12887-019-1461-3

**Published:** 2019-04-12

**Authors:** Fabienne Nackers, Thomas Roederer, Caroline Marquer, Scholastic Ashaba, Samuel Maling, Juliet Mwanga-Amumpaire, Sothara Muny, Chea Sokeo, Vireak Shom, Maria Palha, Pauline Lefèbvre, Beatrice W. Kirubi, Grace Kamidigo, Bruno Falissard, Marie-Rose Moro, Rebecca F. Grais

**Affiliations:** 1 Epicentre, 8 rue Saint Sabin, 75011 Paris, France; 2 Department of Psychiatry, Faculty of Medicine, Mbarara University of Science and Technology, P.O. Box 1410, Mbarara, Uganda; 3 Epicentre, Mbarara Research Centre, P.O. Box 1956, Mbarara, Uganda; 4 Mbarara University of Science and Technology, P.O. Box 1404, Mbarara, Uganda; 5 Medicine Department, Preah Kossamak Hospital, Ministry of Health, Phnom Pen, Cambodia; 6 Médecins Sans Frontières, Phnom Pen, Cambodia; 7 Médecins Sans Frontières, Nairobi, Kenya; 8 Centre de recherche en épidémiologie et santé des populations (CESP)/ Institut national de la santé et de la recherche médicale (INSERM) U1018, Maison de Solenn, Paris, France; 9 Médecins Sans Frontières, Paris, France; 10 Université Paris Descartes, Sorbonne Paris Cité, Hôpital Cochin, Assistance Publique Hôpitaux de Paris, Paris, France

**Keywords:** Mental health, Psychology, Screening, Validation, Preschool children, Low-income population, Kenya, Cambodia, Uganda

## Abstract

**Background:**

In low-resource settings, the lack of mental health professionals and cross-culturally validated screening instruments complicates mental health care delivery. This is especially the case for very young children. Here, we aimed to develop and cross-culturally validate a simple and rapid tool, the PSYCa 6–36, that can be administered by non-professionals to screen for psychological difficulties among children aged six to 36 months.

**Methods:**

A primary validation of the PSYCa 6–36 was conducted in Kenya (*n* = 319 children aged 6 to 36 months; 2014), followed by additional validations in Kenya (*n* = 215; 2014) Cambodia (*n* = 189; 2015) and Uganda (*n* = 182; 2016). After informed consent, trained interviewers administered the PSYCa 6–36 to caregivers participating in the study. We assessed the psychometric properties of the PSYCa 6–36 and external validity was assessed by comparing the results of the PSYCa 6–36 against a clinical global impression severity [CGIS] score rated by an independent psychologist after a structured clinical interview with each participant.

**Results:**

The PSYCa 6–36 showed satisfactory psychometric properties (Cronbach’s alpha > 0.60 in Uganda and > 0.70 in Kenya and Cambodia), temporal stability (intra-class correlation coefficient [ICC] > 0.8), and inter-rater reliability (ICC from 0.6 in Uganda to 0.8 in Kenya). Psychologists identified psychological difficulties (CGIS score > 1) in 11 children (5.1%) in Kenya, 13 children (8.7%) in Cambodia and 15 (10.5%) in Uganda, with an area under the receiver operating characteristic curve of 0.65 in Uganda and 0.80 in Kenya and Cambodia.

**Conclusions:**

The PSYCa 6–36 allowed for rapid screening of psychological difficulties among children aged 6 to 36 months among the populations studied. Use of the tool also increased awareness of children’s psychological difficulties and the importance of early recognition to prevent long-term consequences. The PSYCa 6–36 would benefit from further use and validation studies in popula`tions with higher prevalence of psychological difficulties.

**Electronic supplementary material:**

The online version of this article (10.1186/s12887-019-1461-3) contains supplementary material, which is available to authorized users.

## Background

Despite the lack and heterogeneity of existing prevalence data, the burden of mental health problems in children and adolescents is estimated to be as high as 10–20% worldwide [1]. The largest proportion of this burden is located in low-resource countries, where up to half of the population is younger than 15 years [1]. In these countries, childhood psychological difficulties often remain undetected and thus untreated [2], limiting children’s full developmental potential and increasing the risk of later mental health difficulties [1]. In particular, infant and toddler’s mental health is often very low on the list of priorities [3]. In low-resource countries, the provision of mental health care is hampered by the lack of qualified personnel and limited access to health services [4] combined with stigma and poor awareness of psychological difficulties in young children [5, 6]. The absence of easy-to-use and cross-culturally adapted tools to assess mental health in young children further complicates disease burden estimation [7–10] and the delivery of care [7]. Existing screening tools for children younger than three years may focus on specific disorders or symptoms [11, 12]; necessitate a long administration time [13–18]; require highly-trained administrators [19]; and/or have not been cross-culturally validated in low-resource countries [20, 21]. Validating instruments to assess psychological difficulties in young children living in low-resource countries can provide an important tool to identify those in need. Building on the methods used for the cross-cultural validation of a screening tool designed for children aged three to six years [22, 23], we aimed to develop and to cross-culturally validate a screening tool for psychological difficulties among children aged six to 36 months.

## Methods

### Development of the PSYCa 6–36

As a first step in the development of the PSYCa 6–36, an expert panel based on consensus was convened prior to the start of the study. The panel was comprised of eleven experts in the mental health of infants and young children and transcultural psychopathology from France, Senegal, Canada, USA, and Norway. They were asked to individually list the twelve most important items to screen for psychological difficulties in children aged six to 36 months. Responses were compiled by consensus, aiming for a maximum 20 items, or statements, related to emotions and behaviour that would require little (maximum 10%) or no adaptation when used among different populations. The resulting composition of the PSYCa 6–36 is presented in the Table 1. The tool is completed by the caregiver through an interviewer, with the aid of a guideline (Additional file 1), who reads each item. The caregiver is asked to respond to each item considering the previous month and responding “no or not at all”, “sometimes or occasionally”, “often or frequently”. The interviewer rates each item (0, 1 or 2) accordingly and, at the end of administration, computes a total score ranging from zero to 40, with higher scores indicating greater psychological distress and a need of further mental health assessment. An answer is expected for each item and, when necessary, prompted. However, the rating of a specific item can remain missing if the caregiver does not know or does not want to answer. Examples illustrating each item are included in the guideline for the interviewer.

**Table 1:**
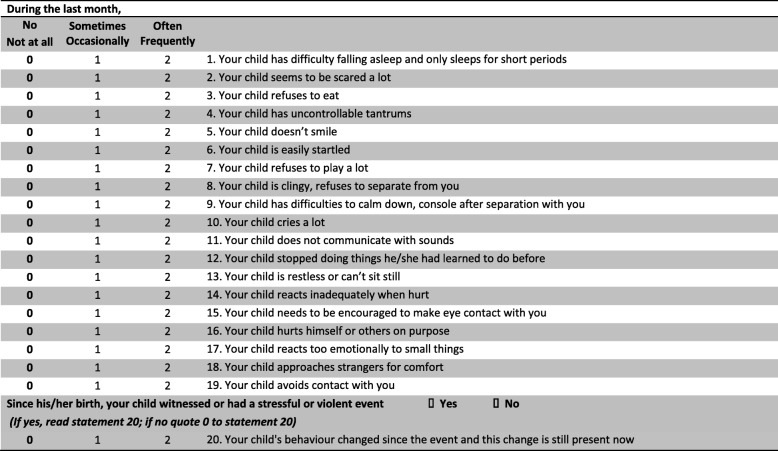
English version of the PSYCa 6–36

### Study setting

The study took place from August 2014 to January 2015 in Mathare, a major urban slum with high level of poverty and violence in Nairobi (Kenya) where Médecins Sans Frontières (MSF) was providing psychological and medical care to victims of sexual violence and to patients diagnosed with multidrug resistant tuberculosis (TB). The study in Mathare was followed by two additional validations. The first took place from July to September 2015 in Kampong Cham, a quiet urban district of Kampong Cham Province (Cambodia) where MSF was providing TB diagnosis, treatment and social support. The second took place from July to August 2016 in Mbarara municipality, the second-largest town of Uganda where Epicentre, a research organisation created by MSF, has been conducting clinical research for over twenty years in collaboration with the Mbarara University of Sciences and Technology (MUST) and the Mbarara Regional Referral Hospital (MRRH). All settings were in low resource but stable environments; none had been affected by a recent acute traumatic event such as a natural disaster or a conflict. MSF and Epicentre facilitated the management, reference and follow-up of children in need of mental health treatment or other relevant medical evaluation and care.

### Translation

Two professional translators fluent in local language (Swahili in Kenya; Khmer in Cambodia; Runyankore in Uganda) and English translated independently the PSYCa 6–36. After reconciliation of the two translations by a mental health professional, the relevance, semantic equivalence and formulation of each item was assessed through discussions with national health professionals, psychosocial workers and groups of caregivers [24]. The resulting translation was back-translated into English. Final translations are presented in the Additional file 1.

### Procedures, population and data collection

Two or three national interviewers were recruited, fluent in the local language and English, in all sites. Children aged six to 36 months accompanied by their main caregiver (child-caregiver dyad) and permanently living in the local community were eligible for participation. Caregivers could be the mother, the father or an adult caring for the child on a regular basis. Children with apparent development retardation or motor disability were not excluded. Exclusion criteria included a previously diagnosed mental health disorder or visible signs of severe mental health disorders. Eligible dyads were selected in the community, starting from the house nearest from a starting point (randomly selected spatial point in Kenya; house randomly selected from a census list in Cambodia; centre of the village in Uganda). Other dyads were recruited by proximity with the objective to include five to eight dyads per day with a maximum of ten per starting point. If several children aged six to 36 months lived in the same house, one was selected at random. Two series of dyads were recruited in Kenya and one series in Cambodia and Uganda.

All children were assessed at home by an interviewer trained to use the PSYCa 6–36. A subsample of children were assessed twice with PSYCa 6–36, 24 hours apart, in the same location, either by a same interviewer to assess the tool’s temporal stability or by different interviewers to assess the tool’s inter-rater reliability. A subsample of children were assessed by a clinical psychologist, blind to results of the PSYCa 6–36. In Kenya, one psychologist worked under the daily supervision of a child psychiatrist experienced in transcultural psychology. In Cambodia and Uganda, a national and an international psychologist assessed most of the children together and otherwise discussed their clinical evaluations. The psychologists were trained by a child psychiatrist to conduct a comprehensive structured mental health examination in young children, through observation and a structured interview with the child’s caregiver. They were also trained to use two additional tools: the Parent-Infant Relationship Global Assessment Scale (PIR-GAS) from the Diagnostic Classification of Mental Health and Developmental Disorders of Infancy and Early Childhood (Revised Edition; DC: 0–3R) [25]; and a seven-point Clinical Global Impression Severity (CGIS) scale assessing the patient’s current symptom(s) severity. The rating of the CGIS scale was considered as the gold standard to assess external validity, with a score higher than one identifying the presence of psychological difficulties.

### Data analysis

Data were double entered in EpiData 3.1 (EpiData, Odense, Denmark) and analysed using Stata (version 13, College Station TX, USA). The total score was calculated as the sum of the individual score for all 20 items. If more than 5 item scores were missing, the total score was not calculated. In case of one to five missing item score(s), the total score was calculated as the sum of the individual item scores and then imputed taking in account the proportion of missing items. Scores were compared between groups using the Kruskal-Wallis test and sensitivity analyses were conducted excluding children with imputed score.

Internal consistency was assessed using Cronbach’s alpha [26] and the inter-rater and temporal stability using the intra-class correlation coefficient (ICC) [27]. Unidimensionality of the instrument was described and different dimensional structures were explored using Catell’s Scree-test [28] and factor analysis with orthogonal varimax. The external validity of the tool, in comparison with the gold standard was assessed using the Spearman’s rho correlation coefficient and using Receiver Operating Characteristic (ROC) curves that plotted the sensitivity against 1–specificity for all PSYCa 6–36 cut-off points to differentiate children with CGIS score of > 1 versus 1. The area under the curve (AUC) were computed with 95% confidence intervals (95% CI), an AUC of 0.5 indicating no discriminating ability, while an AUC of 1.0 indicates perfect discrimination ability.

### Sample size

For the primary validation in Kenya, we aimed to recruit a first series of at least 300 children [29] to estimate a Cronbach’s alpha coefficient with a 95% confidence interval [95%CI] semi-amplitude of 0.05. Of this series, 50 children were assessed twice to estimate the inter-rater reliability and 50 children to assess the temporal stability. In addition, we aimed to recruit a second series of at least 200 children to assess of the external validity. For the subsequent validations conducted in Uganda and Cambodia, a sample of at least 141 children was needed to assess external validity (assuming an AUC under the ROC curve against the CGIS scale of 0.9, with α at 0.05, a power of 0.8, and a standard error ratio between negative and positive results of 0.33), with 20 additional children to assess the inter-rater reliability and 20 children to assess the temporal stability.

### Ethical considerations and consent to participate

Ethical clearance was obtained from the French National Committee for the Protection of Persons (CPP Ile de France XI), the Ethics Review Committee of the Kenyan Medical Research Institute (KEMRI), the Cambodian National Ethics Committee for Health Research (NECHR), the Research Ethics Committee of the Mbarara University of Science and Technology (MUST-REC), and, the Uganda National Council for Science and Technology (UNCST). All participants’ caregivers provided written informed consent before participation. Children in need of psychological or medical care according to the psychologist were offered referral to previously identified professionals for further clinical assessment and, when possible, free treatment.

## Results

In Kenya, 319 children were included in the first series (including 64 assessed twice for the inter-rater reliability and 56 assessed twice for temporal stability) and 215 in the second series. In Cambodia, 148 children were included to assess the external validity; 20 for inter-rater reliability and 21 for temporal stability. In Uganda, 142 children were included to assess external validity; 20 for inter-rater reliability and 20 for temporal stability. None of the children assessed for eligibility presented a previously diagnosed mental health disorder or visible signs of a severe mental health disorder. Participant characteristics are presented in Table 2. Median age of the children included was between 17 and 20 months. Across the three study settings, 19 children had an apparent development retardation or motor disability.

**Table 2:** Participant characteristics, PSYCa 6–36 cross-cultural validation study, Kenya, Cambodia, Uganda

Socio-demographic	Kenya 1 (n = 319)		Kenya 2 (n = 215)		Cambodia (*n* = 148)		Uganda (*n* = 142)	
	n	%	n	%	n	%	n	%
Age of the child (months)								
6–11	77	24.1	58	27.0	26	17.6	31	21.8
12–17	66	20.7	58	27.0	31	21.0	45	31.7
18–23	55	17.2	36	16.7	34	23.0	17	12.0
24–29	70	21.9	28	13.0	26	17.6	24	16.9
30–36	51	16.0	35	16.3	31	21.0	25	17.6
Sex of the child								
Boy	158	49.5	123	57.2	85	57.4	77	54.2
Caregiver-child relation								
Mother	299	93.7	209	97.2	103	69.6	136	95.8
Father	11	3.5	1	0.5	5	3.4	2	1.4
Grandmother	3	1.0	4	1.9	33	22.3	2	1.4
Other (mostly aunt or grandfather)	6	1.9	1	0.5	7	4.7	2	1.4
Household size *(including the child and the caregiver)*								
2 or 3 persons	99	31.0	83	38.6	14	9.5	57	40.1
4 or 5 persons	149	46.7	98	45.6	58	39.2	57	40.1
6 to 13 persons	71	22.3	34	15.8	75	51.4	28	19.8
Number of children < 5 years *(including the child)*								
1	222	69.6	152	70.7	37	25.0	78	54.9
2	92	28.8	54	25.1	54	36.5	53	37.3
3 to 8	5	1.6	9	4.2	57	38.5	11	7.8
Alive siblings from same mother living in same Household								
0	125	39.2	91	42.3	61	41.2	55	38.7
1	95	29.8	69	32.1	47	31.8	35	24.6
2	54	16.9	33	15.4	25	16.9	27	19.1
3 to 5	45	14.1	22	10.2	15	10.1	25	17.6
Parents with which the child usually lives								
Both mother and father	262	82.1	182	84.7	138	93.2	105	73.9
Mother only	51	16.0	29	13.5	4	2.7	33	23.2
Father only	2	0.6	0	0	2	1.4	1	0.7
None	4	1.3	4	1.9	4	2.7	3	2.1
Born ≥1 month preterm	25	7.8	12	5.6	5	3.4	12	8.4
Apparent development retardation or motor disability	9	2.8	1	0.5	3	2.0	6	4.2
Child currently Breastfeeding								
Yes	180	56.4	140	65.1	43	29.1	72	50.7
No	139	43.6	75	34.9	52	35.8	70	49.3
Baby bottle	/	/	/	/	53	35.1	/	/
Child can walk								
Not yet	105	32.9	80	37.2	33	22.3	39	27.6
Since birth, child witness/victim of stressful/violent event^a^	41	12.9	44	20.5	24	16.2	13	9.1

^a^Events reported: Domestic violence (*n* = 48), Fire/burnt (*n* = 9), Accident/injury (*n* = 11), Fighting (*n* = 38), other (*n* = 16)

Due to missing values, 305 (95.6%) PSYCa 6–36 were completed in the first series of Kenya and 145 (98.0%) in Cambodia. There were no missing values in the second series in Kenya or Uganda. The scoring distributions of each item are presented in the Additional file 2. The median total score was a bit lower in the first series in Kenya and in Uganda (Table 3) and there was no evidence for a score difference according to age and sex. The PSYCa 6–36 was administered in a median time less than 15 min (Table 4).

**Table 3:** Total PSYCA 6-36 score for all children, by socio-demographic and clinical characteristics, cross-cultural validation study, Kenya, Cambodia, Uganda

	Kenya 1				Kenya 2				Cambodia				Uganda			
	n	*Median*	IQR*		n	*Median*	IQR*		n	*Median*	IQR*		n	*Median*	IQR*	
All children	319	4^a^	2–8		215	6	3–9		148	5^b^	3–8		142	4	2–8	
		*Min; Max*	*0; 21*			*Min; Max*	*0; 21*			*Min; Max*	*0; 21*			*Min; Max*	*0; 17*	
		*Mean (SD)*	*5.36*	*(4.19)*		*Mean (SD)*	*6.70*	*(4.71)*		*Mean (SD)*	*6.06*	*(3.83)*		*Mean (SD)*	*4.99*	*(3.43)*
	n	*Median*	IQR*	p**	n	*Median*	IQR*	p**	n	*Median*	IQR*	p**	n	*Median*	IQR*	p**
6-12 m	77	4	2–6	0.15	58	7	3–10	0.15	34	7	3–9	0.51	34	4	3–7	0.84
12-24 m	121	5	3–9		94	7	4–10		58	5	3–8		60	5	2.5–7.5	
24-36 m	121	4	2–7		63	5	2–8		56	5	3.5–8		48	5	2–8	
Boys	158	5	3–8	0.70	123	6	3–10	0.62	85	5	3–7	0.41	77	5	3–8	0.12
Girls	160	4	2–8		92	6	3–9		63	6	4–9		65	4	2–7	
CGIS = 1					204	6	3–9	< 0.001	135	5.5	3–8	< 0.001	127	4	2–7	0.02
CGIS> 1					11	11	8–14		13	11	8–16		15	7	3–9	
PIRGAS <81^c^/<91^d^					184	6	3–9	< 0.001	120	5	3–7	0.004	96	4	2–8	0.38
PIRGAS 81^c^/91^d^-100					31	10	7–14		25	8	5–12		46	5	3–7	

*SD* Standard deviation; * *IQR* Interquartile range; ** Kruskal-Wallis test; ^a^ One child with > 25% items missing is excluded from the analysis; 13 children had an incomplete score. Similar results were obtained when excluding children with imputed score (sensitivity analysis); b Three children had an incomplete score due to unknown answers. Similar results were obtained when excluding children with imputed score (sensitivity analysis); c in Cambodia and Uganda; d in Kenya

**Table 4:**
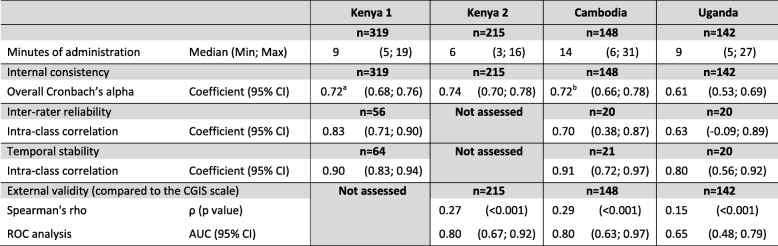
Psychometric properties of the PSYCa 6–36, PSYCa 6–36 cross-cultural validation study, Kenya, Cambodia, Uganda

^a^ including only 305 complete PSYCa 6-36.

^b^ including only 145 complete PSYCa 6-36.

### Internal consistency and reliability

The overall Cronbach’s alpha coefficients were ≥ 0.70 [30, 31], except in Uganda (≥ 0.60) (Table 4). The inter-rater ICC on the total score ranged from 0.63 (Uganda) to 0.83 (in Kenya) and the ICC for temporal stability was ≥0.80 in the three settings.

### External validity

**Fig. 1:**
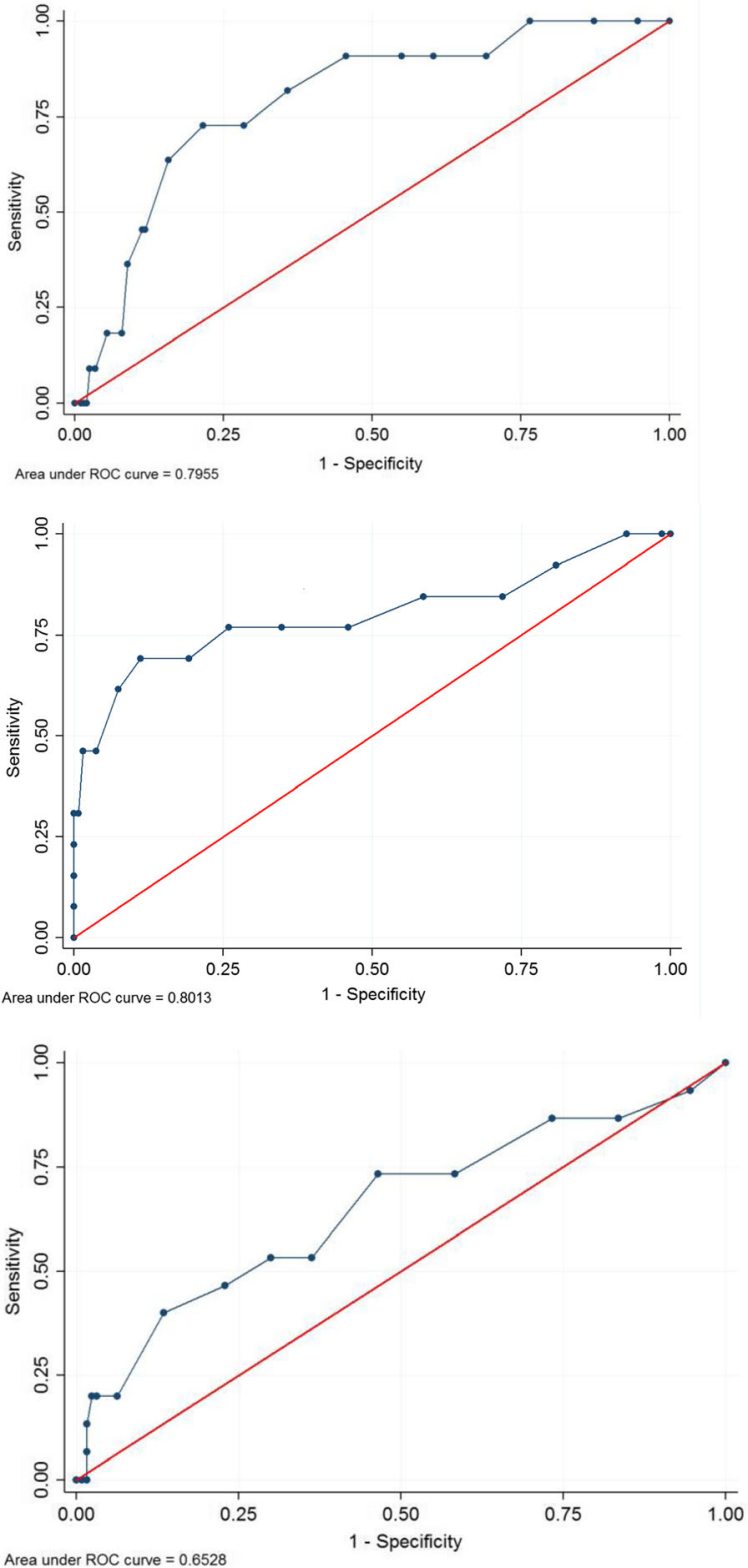
ROC curves of the PSYCA 6–36 score compared with the CGIS score. (Upper: Kenya; Middle: Cambodia; Lower, Uganda)

Psychologists identified difficulties (CGIS score > 1) in 11 (5.1%) children in Kenya, 13 (8.7%) children in Cambodia and 15 (10.5%) in Uganda (Table 5). The distributions of the CGIS and PIR-GAS scores are presented in Table 5. The median PSYCA 6–36 score was higher among children with a CGIS score > 1, and, in Kenya and Cambodia, among children with a lower PIRGAS score (Table 3). The frequency of positive responses per item of the PSYCa 6–36 according to the CGIS score of the children is presented in the Additional file 3. The Spearman’s rho indicated a weak correlation between the final tool and CGIS score (Table 4). The sensitivity and specificity of various PSYCa 6–36 cut-off points to differentiate children with CGIS score of > 1 versus 1 are presented in the Table 6 and the ROC curves in Fig. 1. The area under the ROC curve, measuring the ability of the PSYCa 6–36 to differentiate children with CGIS score of > 1 versus 1, was 0.80 in Kenya and Cambodia but lower in Uganda (Table 4 and Fig. 1). A cut-off point between eight and eleven maximizes the sensitivity and specificity in Kenya and Cambodia but a cut-off point of five is needed to ensure a sensitivity of at least 70% in Uganda. Accounting for the frequency of CGIS score higher than one in the different settings, a cut-off point of eight would identify a third to a fifth (73/215 = 34.0% in Kenya; 35/148 = 23.6% in Cambodia; 29/142 = 20.4% in Uganda) of the total population as falsely positive.

**Table 5:** Clinical evaluation, PSYCa 6–36 cross-cultural validation study, Kenya, Cambodia, Uganda

	Kenya 2 (n = 215)		Cambodia (n = 148)		Uganda (n = 142)	
	n	%	n	%	n	%
CGIS score						
1: Normal, not at all ill	204	94.9	135	91.2	127	89.5
2: Borderline mentally ill	4	1.9	11	7.4	12	8.4
3: Mildly ill	5	2.3	1	0.7	3	2.1
4: Moderately ill	0	0	1	0.7	0	0
5: Markedly ill	1	0.5	0	0	0	0
6: Severely ill	1	0.5	0	0	0	0
7: Extremely ill	0	0	0	0	0	0
PIRGAS scores						
91–100: Well adapted	184	85.6	0	0.0	5	3.6
81–90: Adapted	29	13.5	122	82.4	41	29.3
71–80: Perturbed	2	0.9	20	13.5	65	46.4
61–70: Significantly perturbed	0	0	6	4.1	15	10.7
51–60: Distressed	0	0	0	0	10	7.2
41–50: Disturbed	0	0	0	0	3	2.1
31–40: Disordered	0	0	0	0	1	0.7

^a^2 missing values ^b^ 3 missing values

**Table 6:** Sensitivity and Specificity of various PSYCA 6–36 score cut-off points using CGIS score (> 1 versus 1) as gold standard

PSYCa 6–36 cut-off	CGIS “Not Case” PSYCa 6–36 “Not Case”	CGIS “Case” PSYCa 6–36 “Not Case”	CGIS “Not Case” PSYCa 6–36 “Case”	CGIS “Case” PSYCa 6–36 “Case”	Sensitivity	Specificity	Correctly Classified	LR+	LR-	Positive predictive value	Negative predictive value
	n	n	n	n	%	%	%			%	%
Kenya 2 (n = 215)											
≥ 5	81	1	123	10	90.9	39.7	42.3	1.51	0.23	7.5	98.8
≥ 6	92	1	112	10	90.9	45.1	47.4	1.66	0.20	8.2	98.9
≥ 7	111	1	93	10	90.9	54.4	56.3	1.99	0.17	9.7	99.1
≥ 8	131	2	73	9	81.8	64.2	65.1	2.29	0.28	11.0	98.5
≥ 9	146	3	58	8	72.7	71.6	71.6	2.56	0.38	12.1	98.0
≥ 10	160	3	44	8	72.7	78.4	78.1	3.37	0.35	15.4	98.2
≥ 11	172	4	32	7	63.6	84.3	83.3	4.06	0.43	17.9	97.7
Cambodia (n = 148)											
≥ 5	56	2	79	11	84.6	41.5	45.3	1.45	0.37	12.2	96.6
≥ 6	73	3	62	10	76.9	54.1	56.1	1.67	0.43	13.9	96.1
≥ 7	88	3	47	10	76.9	65.2	66.2	2.21	0.35	17.5	96.7
≥ 8	100	3	35	10	76.9	74.1	74.3	2.97	0.31	22.2	97.1
≥ 9	109	4	26	9	69.2	80.7	79.7	3.59	0.38	25.7	96.5
≥ 10	120	4	15	9	69.2	88.9	87.2	6.23	0.35	37.5	96.8
≥ 11	125	5	10	8	61.5	92.6	89.9	8.31	0.42	44.4	96.2
Uganda (n = 142)											
≥ 5	68	4	59	11	73.3	53.5	55.6	1.58	0.49	15.7	94.4
≥ 6	81	7	46	8	53.3	63.8	62.7	1.47	0.73	14.8	92,0
≥ 7	89	7	38	8	53.3	70.1	68.3	1.78	0.66	17.4	92.7
≥ 8	98	8	29	7	46.7	77.2	73.9	2.04	0.69	19.4	92.5
≥ 9	110	9	17	6	40.0	86.6	81.7	2.98	0.69	26.1	92.4
≥ 10	119	12	8	3	20.0	93.7	85.9	3.17	0.85	27.3	90.8
≥ 11	123	12	4	3	20.0	96.8	88.7	6.35	0.82	42.9	91.1

### Factor analysis and dimensionality

**Fig. 2:**
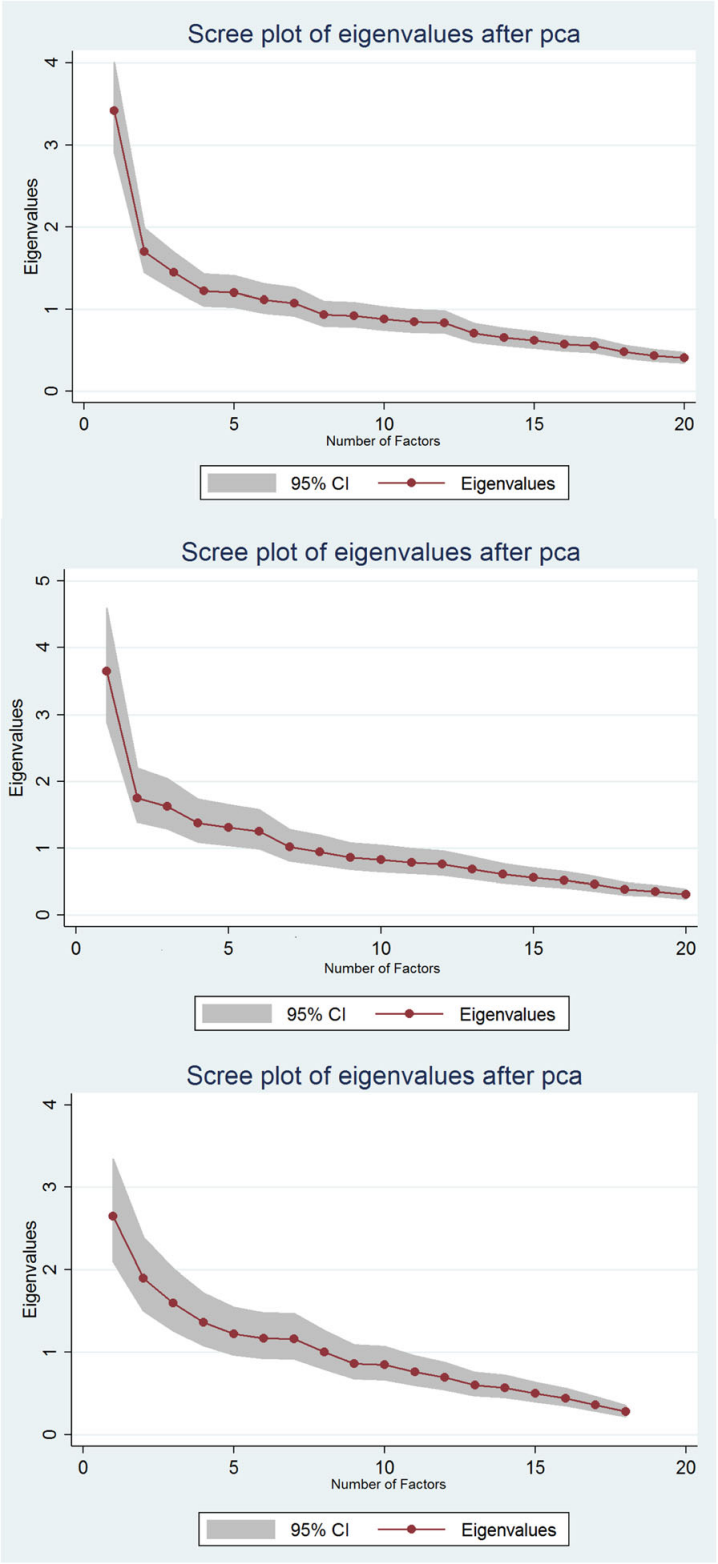
Scree plots of eigenvalues, PSYCa 6–36 cross cultural validation study. (Upper: Kenya; Middle: Cambodia; Lower, Uganda)

The visual exploration of the eigenvalues plot (Cattell’s scree test; Fig. 2) suggests a strong uni-dimensionality in Kenya and Cambodia (one meaningful factor explaining 17 and 18% of the variance) and up to seven factors explaining 61% of the variance in Uganda.

## Discussion

We report the results of a cross-cultural validation study of a new instrument for screening children aged six to 36 months for psychological difficulties. More than 800 children with their caregivers were included across three low-resources settings.

Infant and child psychopathology measurements are challenging, notably due to the rapid motor, cognitive and emotional development in the first three years of life [32, 33]. Considering this complexity, the inherent limitation of quantitative measures to capture human behaviours and emotions [7], as well as the uniqueness and recent development of the PSYCa 6–36, this screening tool showed satisfactory psychometric properties and the ability to classify children with or without psychological difficulties as closely as the CGIS score. The performance of the PSYCa 6–36 was similar in Cambodia and Kenya, two very different cultural and linguistic contexts. This highlights the cross-cultural aspect of the PSYCa 6–36. The performance was lower in Uganda, which might result from actual differences across study populations but also from translation and adaptation flaws [34, 35]. The interviewer guidelines were more frequently used in Uganda than in the two other contexts. A limited comprehension of the items or instructions by the Ugandan participants cannot be excluded. In addition, there were study implementation challenges in Uganda, especially a divergence in the judgments of the psychologists which might have led to suboptimal standardization and clinical assessment. There may also have been administration differences between interviewers. This highlights field constraints and that, despite the ease of use of the PSYCa 6–36, proper training is mandatory.

The PSYCa 6–36 was developed with support of experts in child and transcultural psychopathology and translated by specialists in the local languages and cultural contexts. Translation procedures may not have fully achieved content and sematic equivalence but overall, the PSYCa 6–36 appeared well understood by the participants considering the low frequency (less than 5%) of missing answers. However, some caregivers might have rated some items without full understanding of their meaning or wording, as suggested by some low individual ICC in the test-retest reliability analysis. Also, about 10% of caregivers refused participation and interviewers informally reported that some caregivers felt uncomfortable with the use of quantitative questionnaire and with talking about “abnormal child behaviours” in their household. A lack of awareness of child psychology and the stigma surrounding mental health that affects all populations [4] might have influenced the caregivers’ willingness to disclose information about children’s difficulties. Because of such stigma, caregivers might have provided socially acceptable, consequently biased, answers. A qualitative evaluation might have strengthened the results of this study by shedding light on the caregiver’s perception and acceptance of the use of a questionnaire about child psychology in the different cultures.

For infants and toddlers, direct observation and evaluation of a child interacting with their caregiver in their natural environment remains the best option for mental health assessment [36]. We used the CGIS score assessed by a trained psychologist to assess external validity. The cross-cultural validity of childhood diagnostic criteria in mental health remains debated [9, 37–39]. Although Kenyan, Ugandan and Cambodian psychologists performing the assessment likely limited misinterpretation of possible expressions of mental health disorders or symptoms that may be culture-dependent [9, 37–39]. Further, because of their limited experience in young children’s mental health, they were trained by a child psychiatrist before the start of the study and then worked either under the close supervision or in tandem with a psychiatrist or psychologist experienced in cross-cultural and young child psychology. The clinical assessment was also reinforced by the use of the PIR-GAS scale, although not validated for low-resource settings. Despite these precautions, we recognize the limitation of the comparison with the CGIS might have biased [40] and, possibly underestimated the real PSYCa 6–36 performance.

In Western settings, the prevalence of socio-emotional and behavioural difficulties has been reported to range from 7 to 24% in children aged one to three years [3, 7, 41, 42] but there are data gaps for low-resources countries [1, 4, 8]. A systematic review of prevalence studies of child and adolescent mental health (age range 5 to 16 years) in Sub-Saharan African communities estimated that 14.3% of children had psychopathological difficulties, and 9.5% among studies of which measurement relied on a diagnostic interview [10]. In our study, the psychologists identified fewer children with a CGIS higher than one than expected. Children were included only in the presence of their caregiver and the study was conducted during working hours, thereby likely biasing the study sample towards children at lower risk. More vulnerable children, such as those living in households without a caring adult or left alone during the day, or street children were not included. Also, caregivers who refused participation may be caring for more vulnerable children. Another explanation might be that children living in these difficult environments and exposed to poverty and chronic adversity develop stronger coping mechanisms [7], protecting them against psychological difficulties or limiting the expression of psychological difficulties. This is particularly likely when children remain under the stable protection of their caregiver or other close relatives [7]. Nevertheless, the PSYCa 6–36 would benefit from further use and validation in populations with higher prevalence of psychological difficulties, notably in children having recently faced an acute traumatic event such as migration, conflict, or natural disaster.

In Kenya and Cambodia, the cut-off point maximizing the sensitivity and specificity of the PSYCa 6–36 to differentiate children with CGIS score of > 1 lies between eight and eleven but it is lower in Uganda. Hence, a cut-off point of eight appears an optimal compromise but it should remain flexible to favour sensitivity or specificity according to the expected burden of psychological difficulties and available health services of each specific setting. A cut-off point of eight would identify a substantial proportion of the population as falsely positive, possibly overloading mental health professionals with unnecessary referrals. A higher cut-off would better limit referral to children in need of further clinical evaluation. The definite choice of the cut-off requires subsequent documentation and analysis in populations with higher prevalence of mental health difficulties such as migrants, refugees or internally displaced children, children living in conflict situations or in the aftermath of a natural disaster, or sick children. Further investigation is also needed among specific age groups, such children below one year of age.

In the three study settings, follow-up care was offered by the psychologists and counsellors focusing on the reinforcement of the caregiver-child relationship. Although the child psychiatrists in Kenya and Uganda ensured access to specialized care, such care was limited in Cambodia, being only available in the capital city, a few hours drive from Kampong Cham. It is important to note that, although follow-up care was free of charge, psychologists needed to build trust through repeated home visits to ensure referred children were cared for appropriately. Reducing stigma, misperceptions, and increasing awareness of child psychology among the community and health professionals remain a challenge to support community screening efforts and subsequent access to mental health care [43–46]. The PSYCa 6–36 can also be means to raise awareness of child psychology among the population and of the importance of early recognition to limit long term and developmental consequences.

## Conclusions

The PSYCa 6–36 allowed for rapid screening of psychological difficulties among children aged six to 36 months among the studies populations. Use of the tool also increased awareness of children’s psychological difficulties and the importance of early recognition to prevent long-term consequences. The PSYCa 6–36 would benefit from further use and validation studies in populations with higher prevalence of psychological difficulties.

## Additional files

Additional file 1: PSYCa 6–36 and its guidelines for a standardized administration in English, French, Runyankore, Swahili, and Khmer (PDF 3666 kb)

Additional file 2: Score distribution, missing value, use of the example and Cronbach’s alpha per item of the PSYCa 6–36, cross cultural validation study, Kenya, Cambodia, Uganda. (DOCX 29 kb)

Additional file 3: Frequency of positive responses (Sometimes/occasionally; Often/frequently) per item of the PSYCa 6–36 according to the CGIS score of the children (> 1 versus 1), cross cultural validation study, Kenya, Cambodia, Uganda. (PDF 617 kb)

## Abbreviations

95%CI: 95% confidence interval
AUC: Area under the curve (AUC)
CGIS score: Clinical global impression severity score
CPP: Committee for the Protection of Persons (Ile de France XI)
DC: 0–3R: Diagnostic Classification of Mental Health and Developmental Disorders of Infancy and Early Childhood, Revised Edition
ICC: Intra-class correlation coefficient
KEMRI: Kenyan Medical Research Institute
MSF: Médecins Sans Frontières
MUST: Mbarara University of Science and Technology
NECHR: National Ethics Committee for Health Research
PIR-GAS: Parent-Infant Relationship Global Assessment Scale
PSYCa 6–36: Screening tool for psychological difficulties among children aged six to 36 months
ROC curve: Receiver Operating Characteristic curve
UNCST: Uganda National Council for Science and Technology
