# Supplementary material for: A screening tool for psychological difficulties in children aged 6 to 36 months: cross-cultural validation in Kenya, Cambodia and Uganda
Source: BMC Pediatr. 2019 Apr 12;19:108. doi: 10.1186/s12887-019-1461-3 (PMC6460684; doi:10.1186/s12887-019-1461-3)
Supplement: Supplementary file 1 — PSYCa 6–36 and its guidelines for a standardized administration in English, French, Runyankore, Swahili, and Khmer (PDF 3666 kb) [file 12887_2019_1461_MOESM1_ESM.pdf]

PSYCa 6-36 (English)

This anonymous tool will help improving the understanding of your child's needs and difficulties. Please take your time to think and answer accurately the statements of this tool about your knowledge of your child.

Give the answer that best corresponds to your perception of your child. For each statement, you have three options: no or not at all; sometimes or occasionally; often or frequently. If you do not understand any of the statements, I will give you an example.

For the interviewer: rate 0 for the answer "no or not at all", 1 for "sometimes or occasionally", 2 for "often, frequently". All statements must be answered.

During the last month,

0	1	2	**1. Your child has difficulty falling asleep and only sleeps for short periods**
0	1	2	**2. Your child seems to be scared a lot**
0	1	2	**3. Your child refuses to eat**
0	1	2	**4. Your child has uncontrollable tantrums**
0	1	2	**5. Your child doesn't smile**
0	1	2	**6. Your child is easily startled**
0	1	2	**7. Your child refuses to play a lot**
0	1	2	**8. Your child is clingy, refuses to separate from you**
0	1	2	**9. Your child has difficulties to calm down, console after separation with you**
0	1	2	**10. Your child cries a lot**
0	1	2	**11. Your child does not communicate with sounds**
0	1	2	**12. Your child stopped doing things he/she had learned to do before**
0	1	2	**13. Your child is restless or can't sit still**
0	1	2	**14. Your child reacts inadequately when hurt**
0	1	2	**15. Your child needs to be encouraged to make eye contact with you**
0	1	2	**16. Your child hurts himself or others on purpose**
0	1	2	**17. Your child reacts too emotionally to small things**
0	1	2	**18. Your child approaches strangers for comfort**
0	1	2	**19. Your child avoids contact with you**

Since his/her birth, your child witnessed or had a stressful or violent event

Yes No *(If yes, read statement 20; if no quote 0 to statement 20)*

0 1 2 **20. Your child's behavior changed since the event and this change is still present now**

☐ ☐ ☐ Number of answers per column

☐ ☐ ☐ Scores for each column Overall score: _____

PSYCa 6-36 - Guidelines for a standardized administration

The administration of a screening tool for infant and toddlers requires a rigorous and standardized approach. This guide aims to facilitate the administration of the tool and to assure a standardized administration which is essential to interpret results.

Instructions: It is important to respect the order of the list of statements. The statements are administered one after the other; each statement is quoted 0, 1 or 2.

If a statement seems misunderstood, first, the statement will be repeated once to the respondent, and then the interviewer will use the example.

You must use this example because otherwise the answer would not follow the standard procedures.

1. Your child has difficulty falling asleep and only sleeps for short periods
Repeatedly, over the past month, it has become very difficult to ask to your child to go to bed. For example, your child refuses to lie down in the bed, he/she can also cry and scream. You notice that during the night he/she may also wake up several times and call you.

2. Your child seems to be scared a lot
Over the last month, your child seems disturbed by certain situations and/or things, he/she can even be afraid. The worry concerns situations which do not seem disturbing to you.

3. Your child refuses to eat
Over the last month, your child doesn't want to eat, and/or continuously or repeatedly, does not finish his/her meal even when they are dishes he/she usually enjoyed.

4. Your child has uncontrollable tantrums
Over the last month, when he/she gets angry, the child rolls on the ground by making big gestures or kicking and screaming for example. His/her reactions in certain situations seem to you "excessive" for his/her age compared to other children whom you know.

5. Your child doesn't smile
Over the last month, compared to other children of the same age, you notice that your child doesn't smile often, smiles less, and you notice this even when it is a nice situation, when you talk with him/her or would expect him/her to smile.

6. Your child is easily startled
Over the last month, you notice that your child reacts to specific noises. For example, when a door slams or if there is a metallic noise or if someone just touches him/her from behind, he/she may jump up and be scared.

7. Your child refuses to play a lot
Over the last month, your child has less enthusiasm when it comes to play, even when you ask if he/she wants to play with you or you play with him. You notice for example that compared to other children the same age, playing doesn't interest him/her.

8. Your child is clingy, refuses to separate from you
Over the last month, your child refuses to separate from you, and it is difficult to understand why. If you leave him to do an errand or go buy something for example, he/she can show very strong reactions, which can even be panic, tears, screams, and no one is able to console him/her.

9. Your child has difficulties to calm down, console after separation with you

Over the last month, when you have to leave, your child starts crying and/or screaming, which can last until you come back, during this period, nobody is able to console him/her; he/she will be consoled only if he/she sees you coming back. Also you prefer to go with him/her to avoid this situation.

10. Your child cries a lot

Over the last month, for any reason, your child is crying. Compared to other children the same age, you noticed that the frequency and intensity are very high. As for example, he/she might cry every day, several times a day.

11. Your child does not communicate with sounds

Over the last month, your child doesn't express any sound when interacting with another person, compared to other children of the same age.

12. Your child stopped doing things he had learned to do before

Over the last month, for example, before he/she was able to stand up, and now it is as if he/she can't or he/she started saying a few words but now he/she stopped.

13. Your child is restless or can't sit still

Over the last month, your child moves all the time. He/she does not like to stay in place, and is always agitated. You have the impression that it is more pronounced for him/her compared to other children of the same age.

14. Your child reacts inadequately when hurt

Over the last month, when for example your child falls down, he starts crying a lot, you notice that compared to other children of the same age, he/she can be overreacting or underreacting.

15. Your child needs to be encouraged to make eye contact with you

Over the last month, when you communicate with your child, you notice that often he/she doesn't look at you, as if he/she was avoiding looking at you, to do so often you have to encourage him/her to look at you, in your eyes.

16. Your child hurts himself or others on purpose

Over the last month, your child tends to kick him/herself or others, when he/she does not obtain what he/she wants for example, or when he/she does not agree with you or others. For example, he/she can hit or even bite you or others when he/she doesn't agree.

17. Your child reacts too emotionally to small things

Over the last month, the reactions of your child in certain situations seem to you "excessive" for his/her age compared to other children of the same age whom you know. For example, he/she might look sad, not interacting with others, or hitting, biting him/herself or others, or throwing things.

18. Your child approaches strangers for comfort

Over the last month, you notice that when something happened to your child, when he/she hurts him/herself or a sibling hurts him/her, he/she will approach strangers to be consoled instead of someone he/she knows.

19. Your child avoids contact with you

Over the last month, you notice that your child tends to withdraw from you, that his/her interaction (vocally, eyes, physically) has decreased with you, as if he/she was avoiding it.

Since his/her birth, your child witnessed or had a stressful or violent event

☐ Yes ☐ No

Definition of a stressful and violent event (DSM V) *"Exposure to death, threatened death, actual or threatened serious injury, or actual or threatened sexual violence in one (or more) of the following ways: 1) Direct exposure, 2) Witnessing in person, 3) Indirectly, by learning that a close relative or close friend was exposed to trauma. If the event involved actual or threatened death, it must have been violent or accidental".* This does not include indirect exposure through electronic media, television, movies, or pictures. (Example of event: witnessing interpersonal violence, motor vehicle accidents, experiences of natural disasters, conditions of war, dog bites, invasive medical procedures)

(If yes, read statement 20; if no quote 0 to statement 20)

20. Your child's behavior changed since the event and this change is still present now

Your child lived through a difficult event and since this day, you notice that his/her behavior changed. For example he/she doesn't interact at all or less with others (also you) as he/she did previously, he/she withdraws, he/she became more aggressive.

PSYCa 6-36 (French)

Cet outil anonyme permettra de mieux comprendre les besoins et les difficultés de votre enfant. Prenez votre temps pour répondre en fonction de votre connaissance de votre enfant. Pour chaque item, donnez la réponse qui vous semble la mieux correspondre à votre enfant.

Pour chaque item, vous avez trois possibilités : « non ou pas du tout » ; « parfois ou quelques fois » ; « souvent ou fréquemment ». Si vous ne comprenez pas un des items, je vous donnerai un exemple.

Pour l'interviewer: Vous devez entourer un seul chiffre par item: 0 pour une réponse par la négative ou « pas du tout », 1 « parfois ou quelques fois », 2 « souvent ou fréquemment». Il est nécessaire de répondre à tous les items.

Au cours de ce dernier mois,

0	1	2	**1. Votre enfant a des difficultés pour s'endormir et dort seulement de courtes périodes**
0	1	2	**2. Votre enfant semble effrayé souvent**
0	1	2	**3. Votre enfant refuse de manger**
0	1	2	**4. Votre enfant a des crises de colères incontrôlables**
0	1	2	**5. Votre enfant ne sourit pas**
0	1	2	**6. Votre enfant sursaute facilement**
0	1	2	**7. Votre enfant refuse de jouer souvent**
0	1	2	**8. Votre enfant est collant, il/elle refuse de se séparer de vous**
0	1	2	**9. Votre enfant a du mal à se calmer, à être consolé après être séparé de vous**
0	1	2	**10. Votre enfant pleure beaucoup**
0	1	2	**11. Votre enfant ne communique pas avec des sons**
0	1	2	**12. Votre enfant a arrêté de faire des choses qu'il/elle avait apprises auparavant**
0	1	2	**13. Votre enfant est agité, ne tient pas en place**
0	1	2	**14. Votre enfant réagit de manière adéquate quand il/elle est blessé(e)**
0	1	2	**15. Votre enfant a besoin d'être encourage pour entrer en contact visuel avec vous**
0	1	2	**16. Votre enfant se blesse ou blesse les autres exprès**
0	1	2	**17. Votre enfant réagit trop émotionnellement à des petites choses**
0	1	2	**18. Votre enfant s'approche d'étrangers pour être réconforté**
0	1	2	**19. Votre enfant évite les contacts avec vous**

Depuis sa naissance, votre enfant a été témoin ou a vécu un évènement stressant ou violent

Oui Non *(Si oui, l'item 20 doit être lu ; si non l'item 20 sera coté 0)*

0	1	2	**20. Le comportement de votre enfant a changé depuis cet évènement et ce changement est toujours présent**

☐ ☐ ☐ Nombre de réponses par colonne

☐ ☐ ☐ Score par colonne Score total: _____

PSYCa 6-36 – Guide pour une passation standardisée

L'utilisation d'un outil de dépistage pour des enfants en bas âge requiert une approche rigoureuse et standardisée. Ce guide a pour objectif de faciliter la passation de l'outil et d'assurer une passation standardisée, essentielle à l'interprétation des résultats.

Instructions: Il est important de respecter l'ordre des items. Les items sont posés l'un après l'autre, chaque item est coté 0, 1 ou 2.

Si un item semble mal compris, l'item sera d'abord répété une fois au participant, et seulement après cette répétition, l'enquêteur utilisera l'exemple.

Vous devez utiliser l'exemple proposé sinon la réponse ne répondrait pas aux procédures standardisées.

1. Votre enfant a des difficultés pour s'endormir et dort seulement de courtes périodes
De manière répétée, durant le dernier mois, il est devenu très difficile de demander à votre enfant d'aller au lit. Par exemple, votre enfant refuse de s'allonger dans son lit, il/elle peut également pleurer et hurler. Vous avez remarqué que durant la nuit, il/elle peut aussi se réveiller plusieurs fois et vous appeler.

2. Votre enfant semble effrayé souvent
Durant le dernier mois, votre enfant semble déstabilisé par certaines situations et/ou choses, il/elle peut même en être apeuré. La peur concerne des situations qui ne vous semblent pas inquiétantes.

3. Votre enfant refuse de manger
Durant le dernier mois, votre enfant ne veut pas manger, et/ou de manière continue ou répétée, il/elle ne finit pas son repas même si c'est un plat qu'il apprécie d'habitude.

4. Votre enfant a des crises de colères incontrôlables
Durant le dernier mois, quand il/elle se met en colère, il/elle se roule par terre en faisant des grands gestes ou en tapant et hurlant par exemple. Ses réactions dans certaines situations vous paraissent excessives pour son âge en comparant avec les autres enfants que vous connaissez.

5. Votre enfant ne sourit pas
Durant le dernier mois, en comparaison avec d'autres enfants du même âge, vous avez noté que votre enfant ne souriait pas souvent, souriait moins, et vous avez remarqué cela même lorsqu'il s'agissait de situation agréable, lorsque vous lui parliez ou lorsque vous vous attendiez à ce qu'il/elle sourie.

6. Votre enfant sursaute facilement
Durant le dernier mois, vous avez remarqué que votre enfant réagit à des bruits particuliers. Par exemple, quand une porte claque ou s'il y a un bruit métallique ou si quelqu'un le touche dans le dos, il/elle peut sursauter et avoir peur.

7. Votre enfant refuse de jouer souvent
Durant le dernier mois, votre enfant a moins d'enthousiasme lorsqu'il/elle joue, même lorsque vous lui proposez de jouer avec vous ou lorsque vous jouez avec lui/elle. Vous avez remarqué – par exemple - que, par rapport aux autres enfants du même âge, jouer ne l'intéresse pas.

8. Votre enfant est collant, il/elle refuse de se séparer de vous
Durant le dernier mois, votre enfant refuse de se séparer de vous, et c'est difficile de comprendre pourquoi. Si vous le/la laissez pour faire une course ou bien lui demandez d'aller acheter quelque chose, il/elle peut exprimer une réaction très forte, qui peut même se présenter comme de la panique, des larmes, des cris et personne n'est capable de la/le consoler

9. Votre enfant a du mal à se calmer, à être consolé après être séparé de vous
Durant le dernier mois, quand vous devez partir, votre enfant commence à crier ou à hurler, ce qui peut durer jusqu'à votre retour. Pendant cette période, personne n'arrive à le consoler, il/elle le sera seulement s'il/elle vous voit revenir. Vous préférez partir avec lui/elle pour éviter cette situation.

10. Votre enfant pleure beaucoup
Durant le dernier mois, pour n'importe quelle raison, votre enfant pleure. Comparé aux enfants du même âge, vous avez remarqué que la fréquence et l'intensité est plus importante. Comme par exemple, il/elle peut pleurer chaque jour, plusieurs fois par jour.

11. Votre enfant ne communique pas avec des sons
Durant le dernier mois, votre enfant ne prononce aucun son quand il interagit avec une autre personne, en comparaison avec d'autres enfants du même âge.

12. Votre enfant a arrêté de faire des choses qu'il avait apprises auparavant
Durant le dernier mois, par exemple, alors qu'avant il/elle arrivait à se lever, maintenant c'est comme s'il/elle ne pouvait plus ; ou bien, il/elle avait commencé à dire quelques mots mais maintenant s'est arrêté.

13. Votre enfant est agité, ne tient pas en place
Durant le dernier mois, votre enfant bouge tout le temps. Il/elle n'aime pas rester tranquille, et est toujours agité. Vous avez l'impression que c'est plus prononcé chez lui/elle comparativement à d'autres enfants du même âge.

14. Votre enfant ne réagit de manière adéquate quand il/elle est blessé(e)
Durant le dernier mois, quand par exemple votre enfant tombe, il/elle commence à pleurer beaucoup, vous avez remarqué que, en comparaison à d'autres enfants du même âge, il/elle peut réagir de façon excessive ou insuffisante.

15. Votre enfant a besoin d'être encouragé pour entrer en contact visuel avec vous
Durant le dernier mois, lorsque vous communiquez avec votre enfant, vous avez remarqué que, souvent, il ne vous regarde pas, comme s'il vous évitait. Vous avez besoin de l'encourager pour qu'il/elle vous regarde, dans les yeux.

16. Votre enfant se blesse ou blesse les autres exprès
Durant le dernier mois, votre enfant a tendance à se frapper ou frapper les autres, quand il n'obtient pas ce qu'il veut par exemple ; ou quand il/elle n'est pas d'accord avec vous ou quelqu'un d'autre. Par exemple, il peut frapper ou même mordre quand il n'est pas d'accord.

17. Votre enfant réagit trop émotionnellement à des petites choses
Durant le dernier mois, les réactions de votre enfant dans certaines situations vous semblent excessives pour son âge, comparativement aux enfants du même âge que lui/elle que vous connaissez. Par exemple, il/elle a l'air triste, n'interagit pas avec les autres, ou se tape, se mord ou frappe et mord les autres, ou jette les choses.

18. Votre enfant s'approche d'étrangers pour être réconforté
Durant le dernier mois, vous avez remarqué que quand quelque chose arrive à votre enfant, quand il/elle se fait mal ou son frère ou sa sœur le/la tape, il/elle va demander à un étranger de le/la consoler au lieu de quelqu'un qu'il/elle connaît.

19. Votre enfant évite les contacts avec vous
Durant le dernier mois, vous avez remarqué que votre enfant à tendance s'éloigner de vous, que son interaction (verbale, physique, le regard) a diminué avec vous, comme s'il/elle l'évitait.

Depuis sa naissance, votre enfant a été témoin ou a vécu un événement stressant ou violent

☐ Oui ☐ Non

Définition d'un événement stressant ou violent (DSM5) : « Exposition à la mort, à des blessures graves, ou à la violence sexuelle, effectives ou potentielles, d'une (ou plusieurs) des façons suivantes: 1) Vivre directement l'événement traumatique, 2) Être témoin, en personne, de l'événement vécu par d'autres, 3) Apprendre que l'événement traumatique a été vécu par un membre de la famille proche ou un ami proche. Note : En cas de décès ou de danger de décès d'un membre de la famille ou d'un ami, l'événement doit avoir été violent ou accidentel. Cela ne s'applique pas à l'exposition par le biais des médias électroniques, de la télévision, de films ou de photos. (Exemple d'événement: assister à une scène de violence entre plusieurs personnes, accidents de la circulation, vivre une catastrophe naturelle, guerre, morsures de chien, procédures médicales invasives).

(Si oui, l'item 20 doit être lu ; si non l'item 20 sera coté 0)

20. Le comportement de votre enfant a changé depuis cet évènement et ce changement est toujours présent

Votre enfant a vécu un évenement difficile et depuis lors, vous constatez que son comportement a changé. Par exemple, il/elle n'interagit plus ou peu avec les autres (vous y compris) comme il/elle le faisait avant, il/elle se met à l'écart, il/elle est devenu plus agressif.

epicentre ÉPIDÉMIOLOGIE • EPIDEMIOLOGY   MEDECINS SANS FRONTIERES

PSYCa 6-36 (Runyankore)

Ekihandiiko ky'ebibuuzo ebi nikiza kuhwera omu kwetegyereza ebyetengo n'oburemeezi bw'omwana waawe. Noohaburwa kutwara obwire bwawe, oteekateekye kandi ogarukyemu omu buryo buhikire ebibuuzo ebiri omu kihandiiko eki ebirikukwata aha ku orikwetegyereza omwana waawe.

Garukamu nk'oku orikwetegyereza omwana waawe. Buri kibuuzo oine ebintu bishatu by'okutooranamu: ngaaha; obumwe n'obumwe nari emirundi mingi. Ku oraabe oteetegyereize kimwe aha bibuuzo, ninkuha eky'okureeberaho.

Ahabwa kibuuza: taho 0 ahabw'ekigarukwamu 'ngaaha' ; 1 ahabwa 'obumwe'n'obumwe nari, 2 ahabwa 'emirundi mingi'. Ebibuuzo byona n'eby'okugarukwamu.

Omu kwezi okuhweire

0	1	2	**1. Omwana waawe naagumirwa kugwejegyera nari agwejegyeraho akaire kakye**
0	1	2	**2. Omwana waawe nakira kukangarana**
0	1	2	**3. Omwana waawe naayanga kurya**
0	1	2	**4. Omwana waawe nayoreka ekiniga ekirengire**
0	1	2	**5. Omwana waawe takumwenya**
0	1	2	**6. Omwana waawe naarahuka kuhuruutuka omutima**
0	1	2	**7. Omwana waawe naayanga kuzaana**
0	1	2	**8. Omwana waawe nakira kukuhamiraho atakwenda kukurugaho**
0	1	2	**9. Omwana waawe nagumirwa kucureera ninga kuhuumurizibwa yaheza kukurugaho**
0	1	2	**10. Omwana waawe naarira munonga**
0	1	2	**11. Omwana waawe tarikugambisa eiraka kwenda kuhurizana n'abandi**
0	1	2	**12. Omwana waawe akareka kukora ebi yaabaire yaayegire kukora enyimaho**
0	1	2	**13. Omwana waawe tarikutuuza nari kushutama hamwe**
0	1	2	**14. Omwana waawe tarikutwaza nk'oku kyakubaire yaheza kugubwa kubi**
0	1	2	**15. Omwana waawe naayetenga kuhangaanwa kureebana naiwe maisho-na-maisho**
0	1	2	**16. Omwana waawe naayegwisa kubi nari abandi akigyendereire**
0	1	2	**17. Omwana waawe naarahuka kubakira/ ashemererwa ekirengire ahabw'akantu kakye**
0	1	2	**18. Omwana waawe naahikirira abantu abatamanyirire kumuhumuriza**
0	1	2	**19. Omwana waawe tarikwenda ngu muhikaane**

Kwiha azaarwa, omwana waawe yaareebire nari yaahikirweho ekikorwa kibi/ ebikorwa bibi by'okumutuntuza Eego Ngaaha

(Yaaba 'Eego', buuza ekya 20 yaaba 'Ngaaha', juriza 0 kuhika ahari 20)

0 1 2 20. **Emitwarize y'omwana waawe ekahindukaho kwiha obwo, kandi empinduka egyo na hati ekiriho**

☐ ☐ ☐ Number of answers per column

☐ ☐ ☐ Scores for each column Overall score: _____

Screening for psychological difficulties in children aged 6 to 36 months: cross-cultural validation in Kenya, Cambodia and Uganda.

PSYCa 6-36 – Endagiiriro y'okureetaho obwinganiza omu nyejunisa y'ekikwato

Enyejunisa y'ekikwato eki ky'okushwijuma abeereere n'encuukye neeyetengyesa okwegyendesereza n'obwinganiza. Endagiiriro egi egyendereire kuhwera omu kwejunisa ekikwato, kandi n'okureeba ngu haababo obwinganiza omu kukyejunisa; ekintu ekirikwetengyerwa kimwe kubaasa kushoboorora ebyarugamu.

Okuragiirira

Ni kikuru kugyenda n'oku ebintu bikuratanisiibwa. Ebibuuzo nibibuuzibwa kimwe kikurata ekindi; buri kibuuzo nikijurizibwa 0, 1 nari 2.

Ekibuuzo kyashusha ekitaayetegyerezibwa, nikigarukirwamu kibuuzibwa omurundi ogundi, reero nyakubuuza ayejunisa eky'okureebereho ekihairwe.

Nooragirwa kwejunisa eky'okureeberaho eki, kitari ekyo ekigarukwamu tikiraije kuhikaana n'emitwarize eya buriijo.

1. **Omwana waawe naagumirwa kugwejegyera nari naagwejegyeraho akaire kakye**

Omukwezi okuhweire, kitwire kigumire kugambira omwana waawe kuza kubyama. Nk'eky'okureeberaho, omwana waawe naayanga kubyama, obundi narira kandi ayamuza. Obumwe naimuka emirundi mingi haza akweta.

2. **Omwana waawe nakira kukangarana**

Omukwezi okuhweire, waagyeranisa na buriijo, omwana waawe naashusha orikuteganisibwa *embeera* zitari zimwe, n'obumwe naatiina. Lwe nooshusha oti embeera ezirikumuteganisa tizirikukwerarikiriza.

3. **Omwana waawe naayanga kurya**

Omukwezi okuhweire, omwana waawe nayanga kurya, nari kaingi tarikumaraho byokurya n'obu byakuba eby'arikukunda.

4. **Omwana waawe nayoreka ekiniga ekirengire**

Omukwezi okuhweire, ku arikukwatwa nk'ekiniga, omwana ayekuringa ahansi arikuborooga n'obundi arikushambaguza. Omu mbeera ezimwe, emitwarize ye neekusira erengyesereize ahabw'emyaka ye waamugyeranisa n'abandi baana ab'orikumanya.

5. **Omwana waawe takumwenya**

Omukwezi okuhweire, waamugyeranisa n'abandi baana ab'ekingano kye, nooreeba ngu omwana waawe takukira kumwenya, naamwenya rumwe na rumwe, eki nookireeba n'obu *embeera* erikuba eri nungi okagamba nawe obwo orikuteekateeka ngu abaasa kumwenya.

6. **Omwana waawe naarahuka kuhuruutuka omutima**

Omukwezi okuhweire, obeire nooreeba ngu omwana waawe haine oku arikutwaza omu ruyombo nari embeera endijo. Eky'okureeberaho, ni nk'orwigi waarukinga kifuba rukayomba, nari nk'oruyombo rw'ekyoma, nari omuntu yaamureetwa enyima akamukwataho abaasa kuguruka ahabw'okutiina kandi akangarane.

7. **Omwana waawe naayanga kuzaana**

Omukwezi okuhweire, obeire noreeba ngu omwana waawe aine ekihika kikye aha kuzaana, n'obu waakumushaba kuzaana naiwe. Waamugyeranisa n'abandi baana ab'ekingano kye, nooreeba ngu okuzaana takweteireho.

8. **Omwana waawe naakuhamiraho, tarikwenda kukurugaho**

Omukwezi okuhweire, obeire noreeba ngu omwana waawe tarikwenda kukurugaho, kandi kigumire kumanya n'ahabwenki. Nk'eky'okureeberaho, ku ori kumusigaho, kuza kugira ekiwakora ninga ekiwagura, ayoreka entwaza etari ya buriijo nk'okugira obwoba, okurira, okuteera ekiboroogo kandi tihaine owaakubaasa kumuhuumuruza.

9. Omwana waawe nagumirwa kucureera ninga kuhuumurizibwa yaheza kukurugaho

Omukwezi okuhweire, orebire ngu ku orikwenda kugira ahi waaza, omwana waawe atandika kurira n'okuborooga, n'obumwe aguma atyo mpaka ohotokire, kandi tihaine owaakumurembesereza; naarembeserezibwa yaakureeba wagaruka. Nookunda kugyenda nawe ahabw'okwehara eki.

10. Omwana waawe naarira munonga

Omukwezi okuhweire, orebire ngu omwana waawe naarira ahabw'enshonga yoona. Ku orikumugyeranisa n'abandi baana ab'ekingano kye, oreeba ngu naarengyesereza. Nk'eky'okureeberaho, obumwe naarira buri izooba kandi emirundi mingi.

11. Omwana waawe tarikugambisa eiraka kwenda kuhurizana n'abandi

Omukwezi okuhweire, orebire ngu omwana waawe takugamba na kakye yaaba nahurizana n'ondiijo muntu, wamugyeranisa n'abandi baana ab'ekingano kye.

12. Omwana waawe akareka kukora ebi yaabaire yaayegire kukora enyimaho

Nk'eky'okureeberaho, omukwezi okuhweire, abaire naayemeerera, kwonka hati naashusha nk'otarikubaasa; abaire yaatandikire kwatura ebigambo bikyeho, kwonka hati areraire.

13. Omwana waawe tarikutuuza nari kushutama hamwe

Omukwezi okuhweire, obeire noreeba ngu omwana waawe naagyenda-gyenda obwire bwona. Tarikwenda kuguma hamwe kandi naakira kwimukyeera. Notekateka ngu nikireebwa buri omwe waamugyeranisa n'abandi baana ab'ekingano kye.

14. Omwana waawe tarikutwaza nk'oku kyakubaire yaheza kugubwa/ kugweisibwa kubi

Omukwezi okuhweire, orebire ngu omwana waawe nka hagira ekyamusaasa, narira munonga/ kakye ninga abura kurira, haza ekyo nokyetegyereza gye waagyeranisa n'abandi baana ab'ekingano kye; obumwe naatwaza amaani maingi nari obundi atwaza mpora munoga ahabw'ekyabaho.

15. Omwana waawe naayetenga kuhangaanwa kureebana naiwe maisho-na-maisho

Omukwezi okuhweire, orebire ngu ku orikuteeraho kugaaniira n'omwana waawe, nooreeba ngu kaingi tarikukuhamya maisho, naashusha orikugyezaho kwehara kukureeba. Kaingi nooyetenga kumuhangaana kureebana naiwe maisho na maisho.

16. Omwana waawe naayegwisa kubi nari abandi akigyendereire

Omukwezi okuhweire, omwana waawe naayeteera ninga ateera abandi nka yaaba atatungire eki arikwenda nari atiikirizaine naiwe nari abandi. Nk'eky'okureeberaho, naabaasa kukuteera nari akurume yaaba atiikirizaine naiwe nari shi abandi.

17. Omwana waawe naarahuka kubakira ahabw'akantu kakye

Omukwezi okuhweire, orebire ngu emitwarize y'omwana waawe omu mbeera zitari zimwe neekusira erengyesereize kurugiirira aha myaka ye, waagyeranisa n'abandi baana ab'ekingano kye abu orikumanya. Nk'eky'okureeberaho, obumwe naashusha oine obusaasi, atarikuhikaana na bandi; nari ateera abandi nainga ayeruma, nari ajuguta ebintu oku n'oku.

18. Omwana waawe naahikirira abantu ab'atamanyirire kumuhumuriza

Omukwezi okuhweire, oreebire ngu ku habaire nihagira ekyaba aha mwana waawe, nka yaagubwa kubi nari yagwisa kubi abandi, abaire naahikiirira abantu abatamanyirire kwenda ngu ahuumurizibwe

19. Omwana waawe tarikwenda ngu muhikaane

Omukwezi okuhweire, oreebire ku omwana waawe takwenda ngu muhikaane; ku enkoragana ye naiwe etuubire, naashusha orikugyezaho kukwehara.

Kwiha azaarwa, omwana waawe yaareebire nari yaahikirweho ekikorwa kibi/ ebikorwa bibi by'okumutuntuza ☐ Eego ☐ Ngaaha

Okushoborora ekyabaireho kirikutuntuza kandi ky'obutemu -"Ekyakuhitsya aha kwenda kufa, ekyakutiinisa kukwita, ekyakuhutaaza nari kikatiinisiriza kukuhutaaza, ekyakuhamba nari kikatiinisiriza kukuhamba - ahari gumwe (nari kukiraho) omu miringo egi:<br>*1) Kyakubaho butunu 2) Iwe wenka kukyereebera 3) Kumanyisibwa ku omunyabuzaare nari munywani waawe yaahutaazibwa nari yaatuntuzibwa. Ekyabaho kyaba kirimu okwita nari okutiinisiriza kwita, kihikire kuba kyaba eky'obutemu nari butandu".*<br>Eki tikirimu okuhikwaho ekintu omu buryo bwekiikire nk'okurabira aha mbeho, tereviijoni,ekishushani kya sineema, nari ebishushani.<br>(Eby'okureeberaho ebyakubaho: okureeba ekikorwa ky'obutemu aha gati y'abantu, butandu za motoka, kuhikwaho ebihikiirizi by'obuhangwa, ebyabaho omu ntaro, okurumwa embwa)

(Yaaba 'Eego', buuza ekya 20, yaaba 'Ngaaha', juriza 0 kuhika ahari 20)

20. Entwaza y'omwana waawe ekahindukaho kwiha obwo, kandi empinduka egyo na hati ekiriho

Omwana waawe atwire omu *mbeera* egumire munonga n'okuhitsya hati, nooreeba ngu entwaza ye ehindukire. Nk'eky'okureeberaho, tarikwendera kimwe kukoragana n'abandi (nangwa naiwe) nk'oku abaire naatwaza. Nayenda kwehereera, narahura munonga.

PSYCa 6-36 (Swahili)

Kidadisi hiki kisichotaja jina lolote kitasaidia kuboresha, kuelewa, mahitaji na shida za mtoto wako. Tafadhali fikiria na jibu maswali ya kidadisi kwa makini unapotoa maoni na habari kuhusu mtoto wako.

Toa jibu lilo sahihi kamili kulingana na maoni yako kuhusu mtoto wako. Kwa kila swali, unaweza toa jibu tatu: la, hapana au la hasha; kwa wakati mwingine au mara nyingine; kwa mara kadhaa au mara nyingi. Kama hauelewi swali lolote, naweza kukupa mfano.

Ya Mhojaji: 0 (sufuri) kwa jibu la, hapana au la hasha, 1 (moja) kwa wakati mwingine au mara nyingine, 2 (mbili) kwa mara kadhaa au mara nyingi. Maswali yote lazima yajibiwe.

Kwa mwezi mmoja uliopita,

0	1	2	**1. Mtoto wako ana shida kupata usingizi na analala kwa vipindi vifupi pekee**
0	1	2	**2. Mtoto wako anaonekana kuwa na woga sana**
0	1	2	**3. Mtoto wako anakataa kula**
0	1	2	**4. Mtoto wako anafanya fujo ya kupindukia**
0	1	2	**5. Mtoto wako hatabasamu (smile)**
0	1	2	**6. Mtoto wako anashtuka shtuka kwa urahisi**
0	1	2	**7. Mtoto wako anakataa kucheza sana**
0	1	2	**8. Mtoto wako anakukwamilia wewe, anakataa kutenganishwa na wewe**
0	1	2	**9. Mtoto wako ana shida kutulia au kutulizwa baada ya kutenganishwa na wewe**
0	1	2	**10. Mtoto wako ni wa kulia sana**
0	1	2	**11. Mtoto wako hawasiliani kwa kutumia matamshi au sauti**
0	1	2	**12. Mtoto wako ameacha ujuzi aliyokuwa nayo hapo awali**
0	1	2	**13. Mtoto wako anafadhaika au hawezi kutulia**
0	1	2	**14. Mtoto wako haonyeshi matendo vipasavyo akiumizwa**
0	1	2	**15. Mtoto wako anahitaji kuhimizwa ili kukuangalia machoni**
0	1	2	**16. Mtoto wako anajiumiza mwenyewe au anaumiza wengine kimaksudi**
0	1	2	**17. Mtoto wako anaonyesha hisia kali kwa vitu vidogo vidogo**
0	1	2	**18. Mtoto wako anawaendea watu wengine asiyejua kufarijiwa**
0	1	2	**19. Mtoto wako anakukwepa**

Tangu kuzaliwa, mtoto wako ameshuhudia au amekuwa na kisa cha dhiki (stress) au kisa cha vurugu au vita Ndiyo Hapana/ La

(Ikiwa ni ndiyo uliza swali la 20, ikiwa ni hapana / la weka alama ya 0 kwa swali la 20)

0 1 2 **20. Tabia ya mtoto wako imebadilika tangu kisa hicho na mabadiliko haya bado yanaonekana hadi sasa**

☐ ☐ ☐ Number of answers per column

☐ ☐ ☐ Scores for each column Overall score: _____

PSYCa 6-36 Mwongozo kwa matumizi ya kawaida

Matumizi ya kichujio kwa watoto wachanga na watoto wadogo wanaotambaa yanahitaji mbinu kali na wa kawaida. Mwongozo huu unanuia kuwezesha matumizi ya kifaa na kuhakikisha matumizi ya kawaida ambayo ni muhimu sana kueleza matokeo.

Maagizo : Ni muhimu kufuatilia taratibu ya orodha. Maswali yanatumika moja baada ya nyingine; kila swali lina 0, 1 au 2.

Swali isipoeleweka vyema, kwanza, swali litarudiwa mara moja kwa mhojajiwa, na kisha mhojaji atatumia mfano ulioandikwa baada ya kila swali hapa.

Lazima utumie mfano huu la sivyo jibu halitafuatilia taratibu za kawaida.

1. **Mtoto wako ana shida kupata usingizi na analala kwa vipindi vifupi pekee**

Mara kadhaa, Kwa mwezi mmoja uliopita, kumekuwa shida kumuuliza mtoto wako kuenda kulala. Kwa mfano, mtoto wako anakataa kulala kitandani, na anaweza kulia pia na kupiga mayowe. Pia unatambua kwamba masaa ya usiku anaweza kuamka mara kadhaa, na kukuita wewe.

2. **Mtoto wako anaonekana kuwa na woga sana**

Katika mwezi mmoja sasa, ukilinganisha na kawaida mtoto wako anatatizwa na hali fulani na / au vitu fulani, pia anaweza kuwa mwoga. Hali yenyewe ya tatizo haionekani ni ya kuogofya kwako.

3. **Mtoto wako anakataa kula**

Katika mwezi mmoja sasa, mtoto wako hataki kula, na/au hamalizi mlo wake mfululizo au kwa marudio hata yakiwa milo anayofurahia kwa kawaida.

4. **Mtoto wako anafanya fujo ya kupindukia**

Katika mwezi mmoja sasa, akiwa amekasirika, kwa mfano mtoto anajiangusha chini kwa kuashiria kwa ukali, au kwa kupiga piga mateke na kwa kupiga mayowe. Tabia yake katika hali fulani inaoneka ni ya kupita kiasi kukilinganishwa na watoto wengine wa umri wake anaowajua.

5. **Mtoto wako hatabasamu (smile)**

Katika mwezi mmoja sasa, kukilinganishwa na watoto wa umri wake, unagundua kwamba mtoto wako hatabasamu mara nyingi, anatabasamu mara chache na unagundua haya hata kukiwa na hali nzuri, wakati unazungumza naye au wakati unamtarajia kutabasamu.

6. **Mtoto wako anashtuka shtuka kwa urahisi**

Katika mwezi mmoja sasa, unagundua kwamba mtoto wako anaitikia kelele au kisa fulani. Kwa mfano, mlango ukifungwa kwa nguvu au, kelele ya chuma au, mtu kutoka nyuma akimguza anaweza kuruka kwa mshtuko na kushtuliwa.

7. **Mtoto wako anakataa kucheza sana**

Katika mwezi mmoja sasa, mtoto wako ana hamu kidogo ya michezo, hata kukiwa umemuliza ikiwa anataka kucheza nawe au unataka kucheza naye. Unagundua kwa mfano, kwamba kucheza kwake hakumfurahishi kama watoto wengine wa umri wake.

8. **Mtoto wako anakukwamilia wewe, anakataa kutenganishwa na wewe**

Katika mwezi mmoja sasa, mtoto wako anakataa kutenganishwa na wewe na ni shida kuelewa sababu. Kwa mfano ukimwacha kuenda kwa shughuli fulani au kuenda kununua kitu dukani, anaonyesha hisia kali ambazo ni hofu, machozi, mayowe na hakuna mtu anayeweza kumtuliza.

9. Mtoto wako ana shida kutulia au kutulizwabaada ya kutenganishwa na wewe

Katika mwezi mmoja sasa, wakati wa kuondoka, mtoto wako anaanza kulia au anapiga mayowe, yanayoweza kuendelea hadi utakaporudi bila mtu wowote kumtuliza wakati huo wote; na anaweza kutulizwa tu anapokuona umerudi. Wewe unapendelea kuenda pamoja naye kuepuka hali kama hii.

10. Mtoto wako ni wa kulia sana

Katika mwezi mmoja sasa, kwa sababu yoyote, mtoto wako analia kukilinganishwa na watoto wengine wa umri wake, unagundua kwamba idadi ya marudio na ukali ni wa hali ya juu. Kwa mfano analia kila siku na mara kadhaa kwa siku.

11. Mtoto wako hawasiliani kwa kutumia matamshi au sauti

Katika mwezi mmoja sasa, mtoto wako hatoi tamko au sauti lolote anapoingiliana na mtu wowote kukilinganishwa na watoto wengine wa umri wake.

12. Mtoto wako ameacha ujuzi aliyokuwa nayo hapo awali

Katika mwezi mmoja sasa, kwa mfano, hapo awali aliweza kusimama wima na sasa ni kama hawezi au alianza kutamka maneno machache lakini sasa ameacha.

13. Mtoto wako anafadhaika au hawezi kutulia

Katika mwezi mmoja sasa, mtoto wako anasonga songa nyakati zote. Hapendi kuwa katika sehemu moja na daima anatikisa tikisa. Unatambua kwamba kwake ni zaidi kuliko watoto wengine wa umri wake.

14. Mtoto wako haonyeshi matendo vipasavyo akiumizwa

Katika mwezi mmoja sasa, kwa mfano mtoto wako akianguka chini anaangua kilio kikali na unatambua kwamba kukilinganishwa na watoto wengine wa umri wake anaweza kuwa amezidi au anaonyesha hisia chache sana.

15. Mtoto wako anahitaji kuhimizwa ili kukuangalia machoni

Katika mwezi mmoja sasa, unapowasiliana na mtoto wako, unagundua kwamba mara nyingi hakuangalii, na ni kama anakwepa kukuangalia. Mara nyingi unamshawishi akuangalie machoni.

16. Mtoto wako anajiumiza mwenyewe au anaumiza wengine kimaksudi

Katika mwezi mmoja sasa, kwa mfano mtoto wako anajipiga teke au anapiga wengine teke asipopata atakacho, au asipokubaliana na wewe au wengine. Kwa mfano anaweza kukugonga au kukuuma asipokubaliana na wewe au wengine.

17. Mtoto wako anaonyesha hisia kali kwa vitu vidogo vidogo

Katika mwezi mmoja sasa, hisia ya mtoto wako kwa hali fulani inaonekana kuwa imepita kiasi cha umri wake kukilinganishwa na watoto wengine wa umri wake unaowajua. Kwa mfano anaonekana kuwa na huzuni, akataa kuingiana na wengine au ajigonge/agonge wengine, au ajiume/aume wengine, au arushe rushe vitu

18. Mtoto wako anawaendea watu wengine asiyejua kufarijiwa

Katika mwezi mmoja sasa, unagundua kwamba jambo likifanyika mtoto wako, mtoto wako akijiumiza mwenyewe au akiumizwa na nduguye anawaendea watu wengine asiyejua kufarijiwa badala ya kuendea mtu anayejua.

19. Mtoto wako anakukwepa

Katika mwezi mmoja sasa, unagundua kwamba mtoto wako anajiondoa kutoka kwako, kuingiliana kwake (kwa matamko, kwa macho, kwa kimwili) na wewe kumepungua, na ni kama anakukwepa.

Tangu kuzaliwa, mtoto wako ameshuhudia au amekuwa na kisa cha dhiki (stress) au kisa cha vurugu au vita

☐ **Ndiyo** ☐ **Hapana/ La**

Maana ya kisa chaya dhiki na kisa cha vurugu au vita (DSM V) "Kuleta kifo, tishio la mauti, kujeruhiwa au tishio la majeraha kali, vurugu kamili au tishio la dhuluma za kimapenzi kwa hali moja au zaidi zifuatazo; 1) Kushiriki moja kwa moja, 2) Kushuhudia moja kwa moja, 3) Kutokuwako, kwa kujua kwamba jamaa wa karibu au rafiki wa karibu aliathirika na kuwa na kiwewe. Kukiwa kisa kilikuwa ni kifo au tishio la kifo, lazima kulikuwa kwa vita au kwa ajali"

Haya yote hayajumuishi kushiriki kutokuwako kwa kupitia njia za habari za kielektroniki, televisheni, filamu au picha.

(Mfano wa tukio: Kushuhudia vita vya watu, ajali za magari, matukio ya baa la kawaida, hali ya vita, kuumwa na mbwa taratibu za kimatibabu zinazoweza kuleta madhara)

(Ikiwa ni ndiyo, uliza swali la 20, ikiwa ni hapana/la weka alama ya 0 kwa swali la 20)

20. Tabia ya mtoto wako imebadilika tangu kisa hicho na mabadiliko haya bado yanaonekana hadi sasa

Mtoto wako aliyapitia kisa ngumu na tangu siku hiyo, unagundua kwamba tabia yake imebadilika. Kwa mfano haingilii kamwe au anaingiliana na wengine mara chache (nawe pia) kuliko alivyofanya hapo awali, anajiondoa, na akaleta fujo.

PSYCa 6-36 (Khmer)

កម្រងសំណួរ PSYCa 6-36 (កម្រងសំណួរចិត្តសាស្ត្រសម្រាប់ក្មេងអាយុចាប់ពី ៦ខែ ដល់៣៦ខែ)

កម្រងសំណួរអនាមិកនេះ នឹងជួយធ្វើឱ្យយល់ដឹងកាន់តែប្រសើរឡើងអំពីតម្រូវការ និងការលំបាករបស់កូនអ្នក។ សូមចំណាយពេលពិចារណា និងឆ្លើយសំណួរ ដែលមាននៅក្នុងកម្រងសំណួរនេះឱ្យបានត្រឹមត្រូវអំពីអ្វីដែលអ្នកបានដឹងអំពីកូនរបស់អ្នក។

សូមឆ្លើយសំណួរឱ្យបានត្រឹមត្រូវទៅតាមការយល់ដឹងអំពីកូនរបស់អ្នក។ ចំពោះសំណួរនីមួយៗ អ្នកអាចជ្រើសរើសចម្លើយមួយក្នុងចំណោមចម្លើយទាំងបីគឺ៖ ទេ ឬ អត់ទាល់តែសោះ ជួនកាល ឬ ម្តងម្កាល ឬ ជារឿយៗ ឬ ជាញឹកញាប់។ ប្រសិនបើអ្នកមិនយល់នូវសំណួរណាមួយ ខ្ញុំនឹងលើកជាឧទាហរណ៍ជូនអ្នក។

សម្រាប់អ្នកសម្ភាសន៍៖ លេខ០ សម្រាប់ចម្លើយ ទេ ឬ អត់ទាល់តែសោះ រីឯលេខ១ សម្រាប់ជួនកាល ឬ ម្តងម្កាល ហើយ លេខ២ សម្រាប់ជារឿយៗ ឬ ជាញឹកញាប់។ រាល់សំណួរទាំងអស់ត្រូវតែឆ្លើយ។

**អំឡុងខែមុន**			
០	១	២	១- កូនរបស់អ្នកពិបាកដេកលក់ ហើយដេកភ្ញាក់
០	១	២	២- កូនរបស់អ្នកហាក់ដូចជាភ័យខ្លាចច្រើន
០	១	២	៣- កូនរបស់អ្នកមិនព្រមញ៉ាំអាហារ
០	១	២	៤- កូនរបស់អ្នកមានអារម្មណ៍មួម៉ៅមិនអាចគ្រប់គ្រងបាន
០	១	២	៥- កូនរបស់អ្នកមិនញញឹម
០	១	២	៦- កូនរបស់អ្នកងាយភ្ញាក់
០	១	២	៧- កូនរបស់អ្នកមិនលេងច្រើន
០	១	២	៨- កូនរបស់អ្នកតាម ស្អិត មិនព្រម ចេញឆ្ងាយពីអ្នក
០	១	២	៩- កូនរបស់អ្នកពិបាកលួង នៅពេលបែកពីអ្នក
០	១	២	១០- កូនរបស់អ្នកយំច្រើន
០	១	២	១១- កូនរបស់អ្នកមិនសូវទំនាក់ទំនងដោយសំឡេង/និយាយ
០	១	២	១២- កូនរបស់អ្នកឈប់ធ្វើឬមិនអាចធ្វើអ្វីដែលវាធ្លាប់ធ្វើបានពីមុន
០	១	២	១៣- កូនរបស់អ្នករពិស ឬនៅមិនស្ងៀម
០	១	២	១៤- កូនរបស់អ្នកមានប្រតិកម្មមិនប្រក្រតី(មានសកម្មភាពខ្លាំង ឬតិចតួច) នៅពេលមានការឈឺ(ពេលមានការប៉ះទង្គិច)
០	១	២	១៥-កូនរបស់អ្នកមិនសម្លឹងមករកអ្នកលុះត្រាតែលួងឱ្យគាត់សម្លឹងមករកអ្នក
០	១	២	១៦- កូនរបស់អ្នកធ្វើបាបខ្លួនឯង ឬ មានបំណងធ្វើបាបអ្នកដទៃទៀត
០	១	២	១៧- កូនរបស់អ្នកបញ្ចេញអារម្មណ៍ខ្លាំងចំពោះរឿងតូចតាច
០	១	២	១៨- កូនរបស់អ្នកទៅរកអ្នកដែលវាមិនធ្លាប់ស្គាល់ឱ្យលួងលោម
០	១	២	១៩- កូនរបស់អ្នកគេចមុខពីអ្នក

ងពីវាកើតមក កូនរបស់អ្នកបានឃើញ ឬ បានជួបប្រទះហេតុការណ៍តានតឹង ឬ ហេតុការណ៍ហិង្សាណាមួយ រឬទេ ☐បាទ/ចាំ ☐ទេ *(ប្រសិនបើឆ្លើយ "បាទ/ចាំ" ចូរសួរសំណួរទី២០ ប្រសិនបើឆ្លើយ "ទេ" សូមដាក់លេខ 0 សម្រាប់ សំណួរទី២០)*

សិនបើធ្លាប់បានជួបនឹងហេតុការណ៍ខាងលើ តើហេតុការណ៍នោះជាហេតុការណ៍អ្វី? ______________________

០	១	២	២០- អាកប្បកិរិយារបស់កូនអ្នកមានការផ្លាស់ប្តូរចាប់តាំងពីមានហេតុការណ៍នោះកើតឡើង ហើយការផ្លាស់ប្តូរនេះនៅតែបន្តមានរហូតដល់ពេលពេលឥលូវនេះ

☐ ☐ ☐ Number of answers per column

☐ ☐ ☐ Scores for each column Overall score: _____

PSYCa ៦-៣៦ - សៀវភៅណែនាំសម្រាប់ការគ្រប់គ្រងតាមបទដ្ឋាន

ឯកសារស្រាវជ្រាវអំពីទារកនិងកុមារនេះ តម្រូវឱ្យមានវិធីសាស្ត្រល្អិតល្អន់ និងតាមស្តង់ដារ។ សៀវភៅណែនាំនេះមានគោលបំណងសម្របសម្រួលការគ្រប់គ្រងកម្រងសំណួរ និងដើម្បីធានាការគ្រប់គ្រងតាមបទដ្ឋាន ដែលជាមូលដ្ឋានសំខាន់ក្នុងការបកស្រាយលទ្ធផល។

ការណែនាំ

កត្តាសំខាន់គឺត្រូវធ្វើតាមលំដាប់លំដោយនៃ កម្រងសំណួរ។ រាល់សំណួរទាំងអស់ត្រូវបានសួរ ម្តងមួយៗ ហើយសំណួរនីមួយៗ ត្រូវដាក់លេខ ០, ១ ឬ ២។

ប្រសិនបើសំណួរហាក់ដូចជាធ្វើឱ្យមានការយល់ច្រឡំ ដំបូងត្រូវសួរសំណួរនេះម្តងទៀតទៅអ្នកចូលរួម ហើយបន្ទាប់មកអ្នកសម្ភាសន៍នឹងប្រើឧទាហរណ៍ដែលផ្តល់ឱ្យ។

អ្នកត្រូវប្រើឧទាហរណ៍នេះ បើមិនដូច្នោះទេ ចម្លើយនឹងមិនស្របតាមនីតិវិធីស្តង់ដារឡើយ។

១- កូនរបស់អ្នកពិបាកដេកលក់ ហើយដេកភ្ញាក់

អំឡុងខែមុន វាកាន់តែលំបាកក្នុងការឱ្យកូនរបស់អ្នកចូលគេង។ ឧទាហរណ៍ កូនរបស់អ្នកមិនព្រមគេង វាយំ ហើយស្រែកទៀត។ អ្នកក៏អាចសម្គាល់ផងដែរ អំឡុងពេលយប់ វាអាចភ្ញាក់ច្រើនដង ហើយស្រែកហៅអ្នក។

២- កូនរបស់អ្នកហាក់ដូចជាភ័យខ្លាចច្រើន

អំឡុងខែមុន បើប្រៀបធៀបជាមួយពេលធម្មតា កូនរបស់អ្នកហាក់ដូចជាត្រូវរំខានដោយស្ថានភាពមួយចំនួន វាអាចមានអារម្មណ៍ភ័យខ្លាច។ ស្ថានភាពរំខាននេះហាក់ដូចជាមិនរំខានដល់អ្នកឡើយ។

៣- កូនរបស់អ្នកមិនព្រមញ៉ាំអាហារ

អំឡុងខែមុនកូនរបស់អ្នកមិនចង់ញ៉ាំអាហារ ឬ ញ៉ាំអាហារមិនដែលអស់ឡើយ សូម្បីតែម្ហូបដែលគេចូលចិត្តក៏ដោយ។

៤- កូនរបស់អ្នកមានអារម្មណ៍មួម៉ៅមិនអាចគ្រប់គ្រងបាន

អំឡុងខែមុន ឧទាហរណ៍ នៅពេលដែលកូនរបស់អ្នកខឹង វានៀលលើដី ដោយកាយវិកាច្រងេងច្រងាង ឬទាត់ធាក់ និងស្រែកយំជាដើម។ ប្រតិកម្មរបស់វានៅក្នុងស្ថានភាពមួយចំនួនហាក់ដូចជាធ្វើឱ្យអ្នកគិតថា វាលើសពីវ័យរបស់វា បើប្រៀបធៀបទៅនឹងក្មេងដទៃទៀតដែលអ្នកស្គាល់។

៥- កូនរបស់អ្នកមិនញញឹម

អំឡុងខែមុន បើប្រៀបធៀបទៅនឹងក្មេងដទៃដែលមានអាយុស្របាលគ្នា អ្នកសម្គាល់ឃើញថា ជារឿយៗកូនរបស់អ្នកមិនញញឹម កម្រញញឹម នៅពេលអ្នកនិយាយជាមួយកូន ឬ រំពឹងថាវានឹងញញឹម ហើយអ្នកសម្គាល់ឃើញថាបញ្ហានេះកើតឡើងសូម្បីនៅក្នុងស្ថានភាពល្អក៏ដោយ។

៦- កូនរបស់អ្នកខាយភ្ញាក់

អំឡុងខែមុន អ្នកសម្គាល់ឃើញថា កូនរបស់អ្នកប្រតិកម្មចំពោះសំឡេងរំខាន ឬស្ថានភាពជាក់លាក់ណាមួយ។ ឧទហរណ៍ នៅពេលបិទទ្វារខ្លាំង ឬ ប្រសិនបើមានសំឡេងលោហៈទង្គិចខ្លាំង ឬ ប្រសិនបើនរណាម្នាក់ប៉ះវាពីក្រោយជាដើម កូនរបស់អ្នកអាចស្ទុះលោតឡើងដោយភ្ញាក់ ហើយភ័យខ្លាច។

៧- កូនរបស់អ្នកមិនលេងច្រើន

អំឡុងខែមុន កូនរបស់អ្នកមិនសូវសប្បាយចិត្តនៅពេលវាលេង សូម្បីនៅពេលអ្នកសួរថា តើវាចង់លេងជាមួយអ្នក ឬ អ្នកលេងជាមួយវាក៏ដោយ។ ឧទាហរណ៍ អ្នកសម្គាល់ឃើញថា បើប្រៀបធៀបទៅក្មេងដទៃដែលមានវ័យស្របាលគ្នា វាមិនចាប់អារម្មណ៍នឹងការលេង ឡើយ។

៨- កូនរបស់អ្នកតាម ស្អិត មិនព្រម ចេញឆ្ងាយពីអ្នក

អំឡុងខែមុន កូនរបស់អ្នកមិនព្រមបែកពីអ្នក ហើយក៏មិនដឹងពីមូលហេតុអ្វីឡើយ។ ឧទាហរណ៍ ប្រសិនបើអ្នកទុកឱ្យវាធ្វើអ្វីមួយរយៈពេលខ្លី ឬទៅទិញអ្វីមួយ កូនរបស់អ្នកអាចបង្ហាញប្រតិកម្មខ្លាំង ដូចជា ភ័យតក់ស្លុត ភ័យខ្លាច ស្រែក ហើយគ្មាននរណាម្នាក់អាចលួងលោមបាន។

៩- កូនរបស់អ្នកពិបាកលួង នៅពេលបែកពីអ្នក

អំឡុងខែមុន នៅពេលអ្នកត្រូវចាកចេញ កូនរបស់អ្នកចាប់ផ្តើមយំ និង/ឬស្រែក ដែលអាចរហូតដល់អ្នកត្រឡប់មកវិញទើបបានវាឈប់ ហើយគ្មាននរណាម្នាក់អាចលួងវាបានឡើយទាល់តែឃើញអ្នកត្រឡប់មកវិញ។ ដូច្នេះ អ្នកយកកូនទៅជាមួយដើម្បីចៀសវាងស្ថានភាពបែបនេះ។

១០- កូនរបស់អ្នកយំច្រើន

អំឡុងខែមុន កូនរបស់អ្នកចេះតែយំគ្រប់រឿងទាំងអស់។ បើប្រៀបធៀបទៅនឹងក្មេងដទៃទៀតដែលមានអាយុស្របាលគ្នា អ្នកសម្គាល់ឃើញថា វាកើតឡើងញឹកញាប់ជាច្រើនដង ហើយខ្លាំងៗ។ ឧទាហរណ៍ វាយំរាល់ថ្ងៃ និងច្រើនដងក្នុងមួយថ្ងៃ។

epicentre
ÉPIDÉMIOLOGIE • EPIDEMIOLOGY

MEDECINS SANS FRONTIERES

១១- កូនរបស់អ្នកមិនសូវទំនាក់ទំនងដោយសំឡេង/និយាយ

អំឡុងខែមុន កូនរបស់អ្នកមិនសូវនិយាយស្តីនៅពេលទំនាក់ទំនងជាមួយអ្នកដទៃ បើប្រៀបធៀបទៅនឹងក្មេងដទៃដែលមានអាយុស្របាលគ្នា។

១២- កូនរបស់អ្នកឈប់ធ្វើឬមិនអាចធ្វើអ្វីដែលវាធ្លាប់ធ្វើបានពីមុន

អំឡុងខែមុន ឧទាហរណ៍ពីមុនវាអាចក្រោកឈរ ហើយឥឡូវនេះ ហាក់ដូចជាវាមិនអាច ឬ ពីមុនវាចាប់ផ្តើមនិយាយខ្លះៗ ប៉ុន្តែឥឡូវនេះវាឈប់និយាយ។

១៣- កូនរបស់អ្នករពិស ឬនៅមិនស្ងៀម

អំឡុងខែមុន កូនរបស់អ្នកនៅមិនស្ងៀម។ វាមិនចូលចិត្តនៅមួយកន្លែង ហើយតែងតែពុំស្ងប់ក្នុងចិត្ត។ អ្នកចាប់អារម្មណ៍ថា វាលេចធ្លោជាងគេ បើប្រៀបទៅក្មេងដទៃទៀតដែលមានអាយុស្របាលគ្នា។

១៤- កូនរបស់អ្នកមានប្រតិកម្មមិនប្រក្រតី(មានសកម្មភាពខ្លាំង ឬតិចតួច) នៅពេល ឈឺ (ពេលមានការប៉ះទង្គិច)

ឧទាហរណ៍ អំឡុងខែមុន នៅពេលកូនរបស់អ្នកដួល វាឃំខ្លាំងណាស់ អ្នកសម្គាល់ឃើញថា បើប្រៀបធៀបទៅក្មេងដទៃទៀតដែលមានអាយុស្របាលគ្នា វាអាចមានប្រតិកម្មជ្រុល ឬមិនសូវមានប្រតិកម្ម។

១៥- កូនរបស់អ្នកមិនសម្លឹងមករកអ្នកលុះត្រាតែលួងឱ្យគាត់សម្លឹងមករកអ្នក

អំឡុងខែមុន នៅពេលអ្នកទំនាក់ទំនងជាមួយកូនរបស់អ្នក អ្នកសម្គាល់ឃើញថា ជារឿយៗ កូនរបស់អ្នកមិនសម្លឹងមើលអ្នកទេ ហាក់ដូចជាវាចង់គេចមិនមើលអ្នកអញ្ចឹង។ ជារឿយៗ អ្នកត្រូវជំរុញឱ្យវាសម្លឹងមើលមុខរបស់អ្នក។

១៦- កូនរបស់អ្នកធ្វើបាបខ្លួនឯង ឬ មានបំណងធ្វើបាបអ្នកដទៃទៀត

អំឡុងខែមុន កូនរបស់អ្នកធ្វើបាបខ្លួនឯង ឬ អ្នកដទៃ នៅពេលវាមិនបាននូវអ្វីដែលវាចង់បាន ឬនៅពេលវាមិនយល់ស្របជាមួយអ្នក ឬអ្នកដទៃ។ ឧទាហរណ៍ វាអាចវាយ ឬខាំអ្នក ឬ អ្នកដទៃ នៅពេលវាមិនព្រម។

១៧- កូនរបស់អ្នកបញ្ចេញអារម្មណ៍ខ្លាំងចំពោះរឿងតូចតាច

អំឡុងខែមុន អ្នកសម្គាល់ឃើញថា កូនរបស់អ្នកមានប្រតិកម្ម "ខ្លាំងជ្រុលពេក"សម្រាប់វ័យរបស់វានៅក្នុងស្ថានភាពមួយចំនួន បើប្រៀបធៀបទៅនឹងក្មេងដទៃទៀតអាយុស្របាលគ្នាដែលអ្នកស្គាល់។ ឧទាហរណ៍ វាហាក់ដូចជាស្រងូតស្រងាត់ មិនសូវទំនាក់ទំនងជាមួយអ្នកដទៃ ឬវាយដំខាំខ្លួនឯង ឬ អ្នកដទៃ ឬ គ្រវែងរបស់របរចោលជាដើម។

១៨- កូនរបស់អ្នកទៅរកអ្នកដែលវាមិនធ្លាប់ស្គាល់ឱ្យលួងលោម

អំឡុងខែមុន អ្នកសម្គាល់ឃើញថា នៅពេលមានអ្វីមួយកើតឡើងចំពោះកូនរបស់អ្នក នៅពេលវាធ្វើបាបខ្លួនឯង ឬ បងប្អូនធ្វើបាបវា វាទៅរកមនុស្សប្លែកមុខឱ្យលួងលោម ជំនួសអ្នកដែលវាស្គាល់។

១៩- កូនរបស់អ្នកគេចមុខពីអ្នក

អំឡុងខែមុន អ្នកសម្គាល់ឃើញថា កូនរបស់អ្នកងាកចេញពីអ្នក ហើយវាមិនសូវមានទំនាក់ទំនងជាមួយអ្នក (ការនិយាយស្តី មើលមុខ និងការប៉ះពាល់) ហាក់បីដូចជាវាកំពុងគេចមុខពីអ្នកអញ្ចឹង។

តាំងពីវាកើតមក កូនរបស់អ្នកបានឃើញ ឬ បានជួបប្រទះហេតុការណ៍តានតឹង ឬ ហេតុការណ៍ហិង្សាណាមួយដែរឬទេ ☐បាទ/ចាំ ☐ទេ

និយមន័យអំពីហេតុការណ៍តានតឹង និងហេតុការណ៍ហិង្សា (DSM V) "ប្រឈមការស្លាប់ ការគំរាមសម្លាប់ របួសជាក់ស្តែង ឬ ការគំរាមកំហែងបង្កឲ្យមានរបួសធ្ងន់ធ្ងរ ឬអំពើហិង្សាផ្លូវភេទជាក់ស្តែង ឬការគំរាមកំហែងប្រើហិង្សាលើផ្លូវភេទ តាមរយៈមធ្យោបាយមួយ (ឬ ច្រើន) ក្នុងចំណោមមធ្យោបាយខាងក្រោម៖ ១) ការប្រឈមផ្ទាល់ ២) ការឃើញផ្ទាល់ ៣) ការដឹងដោយប្រយោលថា សាច់ញាតិជិតស្និទ្ធ ឬ មិត្តភក្តិជិតស្និទ្ធប្រឈមនឹងការប៉ះទង្គិចផ្លូវចិត្តធ្ងន់ធ្ងរ។ ប្រសិនបើហេតុការណ៍នោះពាក់ព័ន្ធការសម្លាប់ជាក់ស្តែង ឬការគំរាមសម្លាប់ វាត្រូវជាអំពើហិង្សា ឬ ឧបទ្វេវហេតុ។

នេះមិនរាប់បញ្ចូលការប្រឈមដោយប្រយោលតាមរយៈប្រព័ន្ធផ្សព្វផ្សាយអេឡិចត្រូនិក ទូរទស្សន៍ ភាពយន្ត ឬ រូបភាពឡើយ។
(ឧទាហរណ៍នៃហេតុការណ៍៖ ការឃើញអំពើហិង្សារវាងបុគ្គល គ្រោះថ្នាក់យានយន្ត ការជួបគ្រោះមហន្តរាយធម្មជាតិ ស្ថានភាពសង្គ្រាម ឆ្កែខាំ ការវះកាត់)

(ប្រសិនបើឆ្លើយ "បាទ/ចាំ" ចូរសួរសំណួរទី២០ ប្រសិនបើឆ្លើយ "ទេ" សូមដាក់លេខ 0 សម្រាប់សំណួរទី២០)

២០- អាកប្បកិរិយារបស់កូនអ្នកមានការផ្លាស់ប្តូរចាប់តាំងពីមានហេតុការណ៍នោះកើតឡើង ហើយការផ្លាស់ប្តូរនេះនៅតែបន្តមានរហូតដល់ពេលពេលឥឡូវនេះ

កូនរបស់អ្នករស់នៅឆ្លងកាត់ហេតុការណ៍លំបាក ហើយតាំងពីថ្ងៃនោះមក អ្នកសម្គាល់ឃើញថា អាកប្បកិរិយារបស់វាមានការផ្លាស់ប្តូរ។ ឧទាហរណ៍ វាមិនប្រាស្រ័យទាក់ទងទាល់តែសោះ ឬកម្រទាក់ទងជាមួយអ្នកដទៃ (ក៏ដូចជាអ្នក) ដូចដែលវាធ្លាប់បានធ្វើពីមុន វាគេចឆ្ងាយពីគេ ហើយវាកាន់តែឆេវឆាវ។
