# Supplementary material for: A screening tool for psychological difficulties in children aged 6 to 36 months: cross-cultural validation in Kenya, Cambodia and Uganda
Source: BMC Pediatr. 2019 Apr 12;19:108. doi: 10.1186/s12887-019-1461-3 (PMC6460684; doi:10.1186/s12887-019-1461-3)
Supplement: Supplementary file 2 — Score distribution, missing value, use of the example and Cronbach’s alpha per item of the PSYCa 6–36, cross cultural validation study, Kenya, Cambodia, Uganda. (DOCX 29 kb) [file 12887_2019_1461_MOESM2_ESM.docx]

**Score distribution, missing value, use of the example and Cronbach’s alpha per item of the PSYCa 6-36, cross cultural validation study, Kenya, Cambodia, Uganda.**

| Item | Score = 0 | | Score = 1 | | Score = 2 | | Missing score | | Example used | | Cronbach’s alpha* |
| --- | --- | --- | --- | --- | --- | --- | --- | --- | --- | --- | --- |
|  | n | % | n | % | N | % | n | % | n | % |  |
| **Kenya 1 (n=319 ratings per item)** | | | | | | | | | | | |
| 1 | 214 | 67.1 | 79 | 24.7 | 26 | 8.2 | 0 | 0 | 6 | 1.9 | 0.7036 |
| 2 | 231 | 72.4 | 67 | 21.0 | 20 | 6.3 | 1 | 0.3 | 26 | 8.1 | 0.7003 |
| 3 | 158 | 49.6 | 110 | 34.5 | 51 | 15.9 | 0 | 0 | 1 | 0.3 | 0.7004 |
| 4 | 231 | 72.4 | 59 | 18.5 | 29 | 9.1 | 0 | 0 | 114 | 35.7 | 0.7038 |
| 5 | 303 | 95.0 | 11 | 3.4 | 5 | 1.6 | 0 | 0 | 6 | 1.9 | 0.7010 |
| 6 | 245 | 76.8 | 60 | 18.8 | 14 | 4.4 | 0 | 0 | 13 | 3.9 | 0.6962 |
| 7 | 272 | 85.3 | 42 | 13.1 | 5 | 1.6 | 0 | 0 | 4 | 1.0 | 0.6933 |
| 8 | 160 | 50.2 | 91 | 28.5 | 68 | 21.3 | 0 | 0 | 14 | 4.5 | 0.6981 |
| 9 | 219 | 68.6 | 61 | 19.1 | 39 | 12.3 | 0 | 0 | 26 | 7.8 | 0.6996 |
| 10 | 217 | 68.0 | 70 | 21.9 | 32 | 10.1 | 0 | 0 | 6 | 1.9 | 0.6956 |
| 11 | 311 | 97.5 | 5 | 1.6 | 2 | 0.6 | 1 | 0.3 | 50 | 15.9 | 0.7109 |
| 12 | 300 | 94.0 | 11 | 3.5 | 5 | 1.6 | 3 | 0.9 | 100 | 31.3 | 0.7071 |
| 13 | 249 | 78.1 | 43 | 13.5 | 22 | 6.9 | 5 | 1.6 | 83 | 26.0 | 0.7131 |
| 14 | 294 | 92.1 | 21 | 6.6 | 1 | 0.3 | 3 | 1.0 | 72 | 22.6 | 0.7141 |
| 15 | 284 | 89.0 | 25 | 7.8 | 8 | 2.5 | 2 | 0.6 | 99 | 31.4 | 0.7117 |
| 16 | 210 | 65.8 | 85 | 26.6 | 22 | 6.9 | 2 | 0.6 | 20 | 6.3 | 0.6994 |
| 17 | 243 | 76.2 | 56 | 17.6 | 17 | 5.3 | 3 | 1 | 123 | 38.5 | 0.6968 |
| 18 | 280 | 87.8 | 25 | 7.8 | 12 | 3.8 | 2 | 0.6 | 51 | 16.2 | 0.7188 |
| 19 | 294 | 92.1 | 23 | 7.2 | 1 | 0.3 | 1 | 0.3 | 9 | 2.9 | 0.7125 |
| 20 | 309 | 96.8 | 6 | 1.9 | 3 | 1.0 | 1 | 0.3 | 0 | 0.0 | 0.7262 |
| **Kenya 2 (n=215 ratings per item)** | | | | | | | | | | | |
| 1 | 130 | 60.5 | 57 | 26.5 | 28 | 13.0 | 0 | 0 | 1 | 0.5 | 0.7311 |
| 2 | 149 | 69.3 | 47 | 21.9 | 19 | 8.8 | 0 | 0 | 14 | 6.5 | 0.7332 |
| 3 | 84 | 39.1 | 85 | 39.5 | 46 | 21.4 | 0 | 0 | 0 | 0 | 0.7372 |
| 4 | 149 | 69.3 | 46 | 21.4 | 20 | 9.3 | 0 | 0 | 49 | 22.8 | 0.7188 |
| 5 | 190 | 88.4 | 23 | 10.7 | 2 | 0.9 | 0 | 0 | 0 | 0 | 0.7332 |
| 6 | 161 | 74.9 | 40 | 18.6 | 14 | 6.5 | 0 | 0 | 5 | 2.3 | 0.7320 |
| 7 | 181 | 84.2 | 31 | 14.4 | 3 | 1.4 | 0 | 0 | 0 | 0 | 0.7269 |
| 8 | 98 | 45.6 | 70 | 32.6 | 47 | 21.9 | 0 | 0 | 6 | 2.8 | 0.7381 |
| 9 | 133 | 61.9 | 55 | 25.6 | 27 | 12.6 | 0 | 0 | 4 | 1.9 | 0.7275 |
| 10 | 144 | 67.0 | 50 | 23.3 | 21 | 9.8 | 0 | 0 | 1 | 0.5 | 0.7222 |
| 11 | 206 | 95.8 | 7 | 3.3 | 2 | 0.9 | 0 | 0 | 2 | 0.9 | 0.7378 |
| 12 | 195 | 90.7 | 16 | 7.4 | 4 | 1.9 | 0 | 0 | 55 | 25.6 | 0.7462 |
| 13 | 159 | 74.0 | 29 | 13.5 | 27 | 12.6 | 0 | 0 | 41 | 19.1 | 0.7367 |
| 14 | 193 | 89.8 | 18 | 8.4 | 4 | 1.9 | 0 | 0 | 39 | 18.1 | 0.7352 |
| 15 | 172 | 80.0 | 27 | 12.6 | 16 | 7.4 | 0 | 0 | 43 | 20.0 | 0.7507 |
| 16 | 127 | 59.1 | 72 | 33.5 | 16 | 7.4 | 0 | 0 | 4 | 1.9 | 0.7333 |
| 17 | 143 | 66.5 | 57 | 26.5 | 15 | 7.0 | 0 | 0 | 65 | 30.2 | 0.7332 |
| 18 | 181 | 84.2 | 25 | 11.6 | 9 | 4.2 | 0 | 0 | 31 | 14.4 | 0.7398 |
| 19 | 185 | 86.1 | 27 | 12.6 | 3 | 1.4 | 0 | 0 | 7 | 3.3 | 0.7311 |
| 20 | 205 | 95.4 | 7 | 3.3 | 3 | 1.4 | 0 | 0 | 0 | 0 | 0.7435 |

| Item | Score = 0 | | Score = 1 | | Score = 2 | | Missing score | | Example used | | Cronbach’s alpha* |
| --- | --- | --- | --- | --- | --- | --- | --- | --- | --- | --- | --- |
|  | n | % | n | % | N | % | n | % | n | % |  |
| **Cambodia (n=148 ratings per item)** | | | | | | | | | | | |
| 1 | 94 | 63.5 | 43 | 29.1 | 11 | 7.4 | 0 | 0 | 10 | 6.8 | 0.7052 |
| 2 | 118 | 79.7 | 23 | 15.5 | 7 | 4.7 | 0 | 0 | 6 | 4.1 | 0.7068 |
| 3 | 107 | 72.3 | 39 | 26.4 | 2 | 1.4 | 0 | 0 | 5 | 3.4 | 0.6974 |
| 4 | 95 | 64.2 | 43 | 29.1 | 10 | 6.8 | 0 | 0 | 29 | 19.6 | 0.6934 |
| 5 | 127 | 85.8 | 20 | 13.5 | 1 | 0.7 | 0 | 0 | 1 | 0.7 | 0.7136 |
| 6 | 116 | 78.4 | 29 | 19.6 | 2 | 1.4 | 1 | 0.7 | 61 | 41.2 | 0.7141 |
| 7 | 133 | 89.9 | 13 | 8.8 | 2 | 1.4 | 0 | 0 | 0 | 0 | 0.7136 |
| 8 | 78 | 52.7 | 52 | 35.1 | 18 | 12.2 | 0 | 0 | 12 | 8.1 | 0.6976 |
| 9 | 123 | 83.1 | 20 | 13.5 | 5 | 3.4 | 0 | 0 | 4 | 2.7 | 0.6970 |
| 10 | 83 | 56.1 | 57 | 38.5 | 8 | 5.4 | 0 | 0 | 0 | 0.0 | 0.7029 |
| 11 | 136 | 91.9 | 12 | 8.1 | 0 | 0.0 | 0 | 0 | 27 | 18.2 | 0.7108 |
| 12 | 135 | 91.2 | 11 | 7.4 | 1 | 0.7 | 1 | 0.7 | 67 | 45.3 | 0.7331 |
| 13 | 6 | 4.1 | 59 | 39.9 | 83 | 56.1 | 0 | 0 | 0 | 0.0 | 0.7142 |
| 14 | 136 | 91.9 | 10 | 6.8 | 0 | 0.0 | 2 | 1.4 | 34 | 23.0 | 0.7279 |
| 15 | 139 | 93.9 | 9 | 6.1 | 0 | 0.0 | 0 | 0 | 12 | 8.1 | 0.7264 |
| 16 | 79 | 53.4 | 60 | 40.5 | 9 | 6.1 | 0 | 0 | 9 | 6.1 | 0.6960 |
| 17 | 96 | 64.9 | 41 | 27.7 | 11 | 7.4 | 0 | 0 | 31 | 21.0 | 0.7011 |
| 18 | 143 | 96.6 | 4 | 2.7 | 1 | 0.7 | 0 | 0 | 3 | 2.0 | 0.7348 |
| 19 | 142 | 95.9 | 6 | 4.1 | 0 | 0.0 | 0 | 0 | 3 | 2.0 | 0.7040 |
| 20 | 145 | 98.0 | 2 | 1.4 | 1 | 0.7 | 0 | 0 | 0 | 0.0 | 0.7009 |
| **Uganda (n=142 ratings per item)** | | | | | | | | | | | |
| 1 | 107 | 75.3 | 24 | 16.9 | 11 | 7.8 | 0 | 0 | 5 | 3.5 | 0.5989 |
| 2 | 105 | 73.9 | 26 | 18.3 | 11 | 7.8 | 0 | 0 | 7 | 4.9 | 0.5854 |
| 3 | 98 | 69.0 | 32 | 22.5 | 12 | 8.5 | 0 | 0 | 4 | 2.8 | 0.5897 |
| 4 | 84 | 59.1 | 44 | 31.0 | 14 | 9.9 | 0 | 0 | 26 | 18.3 | 0.5813 |
| 5 | 134 | 94.4 | 8 | 5.6 | 0 | 0 | 0 | 0 | 142 | 100 | 0.5869 |
| 6 | 106 | 74.7 | 28 | 19.7 | 8 | 5.6 | 0 | 0 | 60 | 42.2 | 0.5984 |
| 7 | 142 | 100 | 0 | 0 | 0 | 0 | 0 | 0 | 1 | 0.7 | ** |
| 8 | 79 | 55.6 | 31 | 21.8 | 32 | 22.6 | 0 | 0 | 17 | 11.0 | 0.5815 |
| 9 | 117 | 82.4 | 11 | 7.7 | 14 | 9.9 | 0 | 0 | 22 | 15.5 | 0.5801 |
| 10 | 115 | 81.0 | 13 | 9.1 | 14 | 9.9 | 0 | 0 | 7 | 4.9 | 0.5844 |
| 11 | 142 | 100 | 0 | 0 | 0 | 0 | 0 | 0 | 142 | 100 | ** |
| 12 | 136 | 95.8 | 5 | 3.5 | 1 | 0.7 | 0 | 0 | 66 | 46.5 | 0.6266 |
| 13 | 91 | 64.1 | 26 | 18.3 | 25 | 17.6 | 0 | 0 | 23 | 16.2 | 0.6250 |
| 14 | 120 | 84.5 | 19 | 13.4 | 3 | 2.1 | 0 | 0 | 60 | 42.2 | 0.5764 |
| 15 | 136 | 95.6 | 5 | 3.3 | 1 | 1.1 | 0 | 0 | 142 | 100 | 0.5986 |
| 16 | 103 | 72.5 | 31 | 21.8 | 8 | 5.6 | 0 | 0 | 29 | 20.4 | 0.6093 |
| 17 | 118 | 83.1 | 21 | 14.8 | 3 | 2.2 | 0 | 0 | 34 | 23.9 | 0.5885 |
| 18 | 94 | 66.2 | 35 | 24.6 | 13 | 9.2 | 0 | 0 | 21 | 14.8 | 0.6172 |
| 19 | 141 | 99.3 | 1 | 0.7 | 0 | 0 | 0 | 0 | 142 | 100 | 0.6114 |
| 20 | 134 | 94.3 | 7 | 4.9 | 1 | 0.8 | 0 | 0 | 1 | 0.7 | 0.5974 |

** Alpha coefficient for the scale including all but this item.*

*** Item with constant values, alpha cannot be computed.*
