# Supplementary material for: A screening tool for psychological difficulties in children aged 6 to 36 months: cross-cultural validation in Kenya, Cambodia and Uganda
Source: BMC Pediatr. 2019 Apr 12;19:108. doi: 10.1186/s12887-019-1461-3 (PMC6460684; doi:10.1186/s12887-019-1461-3)
Supplement: Supplementary file 3 — Frequency of positive responses (Sometimes/occasionally; Often/frequently) per item of the PSYCa 6–36 according to the CGIS score of the children (> 1 versus 1), cross cultural validation study, Kenya, Cambodia, Uganda. (PDF 617 kb) [file 12887_2019_1461_MOESM3_ESM.pdf]

Frequency of positive responses (Sometimes/occasionally; Often/frequently) per item of the PSYCa 6-36 according to the CGIS score of the children (>1 versus 1), cross cultural validation study, Kenya, Cambodia, Uganda.

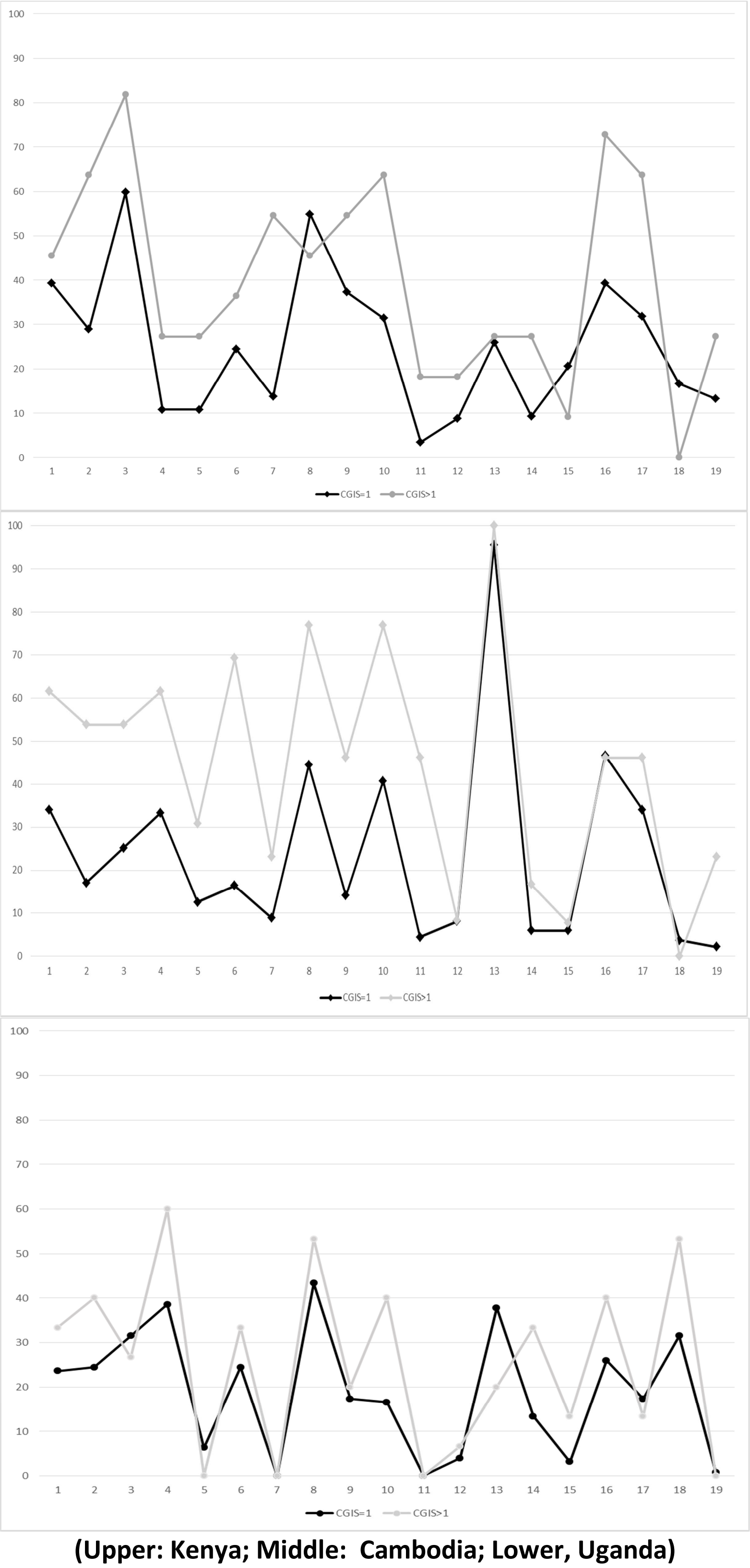

(Upper: Kenya; Middle: Cambodia; Lower, Uganda)
